# Identification of the dehydrin gene family from grapevine species and analysis of their responsiveness to various forms of abiotic and biotic stress

**DOI:** 10.1186/1471-2229-12-140

**Published:** 2012-08-10

**Authors:** Yazhou Yang, Mingyang He, Ziguo Zhu, Shuxiu Li, Yan Xu, Chaohong Zhang, Stacy D Singer, Yuejin Wang

**Affiliations:** 1 College of Horticulture, Northwest A&F University, Yangling, Shaanxi, 712100, China; 2 Key Laboratory of Biology and Genetic Improvement of Horticultural Crops (Northwest Region), Ministry of Agriculture, Northwest A&F University, Yangling, Shaanxi, 712100, China; 3 State Key Laboratory of Crop Stress Biology in Arid Areas, Northwest A&F University, Yangling, Shaanxi, 712100, China; 4 Department of Agricultural, Food and Nutritional Science, 4–10 Agriculture/Forestry Centre, University of Alberta, Edmonton, Alberta, T6G 2P5, Canada

**Keywords:** Grapevine, Dehydrin, Stress-induced expression, Powdery mildew, Promoter

## Abstract

**Background:**

Dehydrins (DHNs) protect plant cells from desiccation damage during environmental stress, and also participate in host resistance to various pathogens. In this study, we aimed to identify and characterize the *DHN* gene families from *Vitis vinifera* and wild *V. yeshanensis*, which is tolerant to both drought and cold, and moderately resistant to powdery mildew.

**Results:**

Four *DHN* genes were identified in both *V. vinifera* and *V. yeshanensis*, which shared a high sequence identity between the two species but little homology between the genes themselves. These genes were designated *DHN1*, *DHN2*, *DHN3* and *DHN4*. All four of the DHN proteins were highly hydrophilic and were predicted to be intrinsically disordered, but they differed in their isoelectric points, kinase selectivities and number of functional motifs. Also, the expression profiles of each gene differed appreciably from one another. Grapevine *DHN1* was not expressed in vegetative tissues under normal growth conditions, but was induced by drought, cold, heat, embryogenesis, as well as the application of abscisic acid (ABA), salicylic acid (SA), and methyl jasmonate (MeJA). It was expressed earlier in *V. yeshanensis* under drought conditions than in *V. vinifera*, and also exhibited a second round of up-regulation in *V. yeshanensi*s following inoculation with *Erysiphe necator*, which was not apparent in *V. vinifera*. Like *DHN1*, *DHN2* was induced by cold, heat, embryogenesis and ABA; however, it exhibited no responsiveness to drought, *E. necator* infection, SA or MeJA, and was also expressed constitutively in vegetative tissues under normal growth conditions. Conversely, *DHN3* was only expressed during seed development at extremely low levels, and *DHN4* was expressed specifically during late embryogenesis. Neither *DHN3* nor *DHN4* exhibited responsiveness to any of the treatments carried out in this study. Interestingly, the presence of particular *cis*-elements within the promoter regions of each gene was positively correlated with their expression profiles.

**Conclusions:**

The grapevine *DHN* family comprises four divergent members. While it is likely that their functions overlap to some extent, it seems that *DHN1* provides the main stress-responsive function. In addition, our results suggest a close relationship between expression patterns, physicochemical properties, and c*is*-regulatory elements in the promoter regions of the *DHN* genes.

## Background

Dehydrins (DHNs) are a class of hydrophilic, thermostable stress proteins with a high number of charged amino acids that belong to the Group II Late Embryogenesis Abundant (LEA) family. Genes that encode these proteins are expressed during late embryogenesis, as well as in vegetative tissues subjected to drought, low temperature and high salt conditions [1-3]. Intriguingly, over-expression of *DHN* genes in transgenic plants has been found to enhance resistance of the transgenic lines to various adverse environments, such as cold, drought, salinity and osmotic stress [4-7], which has raised significant interest in their putative application for crop improvement. Furthermore, it has recently been shown that reduced levels of dehydrins in transgenic Arabidopsis seeds leads to reduced seed longevity [8], emphasizing their importance to seed survival in addition to their influence on vegetative stress tolerance.

While it is generally accepted that DHNs function to protect cells from damage caused by stress-induced dehydration [9], their precise mechanism remains elusive. However, it has been proposed that they may carry out their function through membrane stabilization by acting as chaperones to prevent the aggregation and/or inactivation of proteins under dehydration or high temperature conditions [5,10,11].

Classification of DHNs is based upon structural features of the proteins, such as the presence and copy number of certain conserved motifs, such as the K-, S-, and Y-segments. To date, these proteins have been divided into 5 subclasses, including Y_n_SK_n_, Y_n_K_n_, SK_n_, K_n_ and K_n_S [12]. All DHNs possess at least one K-segment (EKKGIMDKIKEKLPG), which is generally located at the C-terminal end of the protein and has the ability to form an amphipathic helix-like structure that may play a role in its interaction with membranes and proteins [13,14]. The S-segment consists of a track of serines that can be modified through phosphorylation and may function in the regulation of protein conformation and ion-binding activity [15-17]. The Y-segment (DEYGNP) is located near the N-terminus and shows partial amino acid identity to the nucleotide binding site motif of chaperone proteins from various organisms [12].

Several other conserved regions have also been identified in a subset of DHNs. For example, lysine-rich segments (Lys-segments) contain a cluster of lysines that are generally located between the S- and K-segments [18,19] and have been suggested to participate in the binding of DHNs to DNA or RNA [19]. Nuclear localization signals (NLSs), which bear an RRKK motif, have been found specifically in YSK_n_-type DHNs and play a role in their localization to the nucleus [15,16,20]. Furthermore, phosphorylation has been found to be an important factor for substrate binding of DHNs [16,17,21], and recently a His switch has been found to be involved in the regulation of membrane binding of the *Arabidopsis thaliana* DHN, LTI30 [22].

At the functional level, *DHN* family members often exhibit sub-functionalization, with different genes displaying differential expression profiling throughout development and under stress conditions. For example, while both *LTI29* (SK_2_) and *LTI30* (K_6_) were up-regulated in Arabidopsis under low temperature conditions, only *LTI30* was up-regulated following salt treatments [1]. Similarly, while ten barley *DHN*s were found to be up-regulated by drought, only three were up-regulated by low temperatures [23], and in *Oleae europaea*, although expression levels of 40 kDa and 42 kDa *DHN*s increased in response to various stressors (including dehydration, high salinity, and wounding), 16 kDa and 18 kDa *DHN*s were mainly induced by salt stress [24]. These differences in expression patterns imply functional diversification within this gene family; however, at present, the relationship between subgroup classification and expression profile is unclear [9].

Grapevine is one of the most important fruit crops in the world and while the majority of grape varieties are directly cultivated from *Vitis vinifera* L., this species is relatively susceptible to powdery mildew (*Erysiphe necator*). Conversely, *V. yeshanensis* is a wild species of grape native to the Yanshan mountain in Hebei province, China, that is highly tolerant to both cold and drought [25,26], and is also resistant to *E. necator*[27]. Previously, two highly similar putative Y_2_SK_2_-type *DHN* genes (*DHN1a* and *DHN1b*) were identified in *V. vinifera* and their expression was found to be induced by multiple types of stress, such as drought, cold and high salinity [28,29]. In this study, we aimed to identify the members of *DHN* gene family in *V. vinifera*, as well as their homologous equivalents in *V. yeshanensis*. In doing so, we were able to investigate the functional divergence of this gene family in these two species through comparisons of their expression profiles and putative physicochemical characteristics. Furthermore, we also assessed possible relationships between specific *cis*-elements within *DHN* promoter sequences and the regulation of their expression under various conditions.

## Results

### Identification of *DHN* family members in *V. vinifera* and *V. yeshanensis*

A 280-bp fragment of a *DHN* cDNA was cloned from drought-treated leaves of *V. yeshanensis* acc. Yanshan-1 using differential display reverse transcription-PCR (DDRT-PCR; Additional file 1). Subsequently, the full-length sequence was determined using 5’ rapid amplification of cDNA ends (RACE) and was termed *VyDHN1* [GenBank:JF900497]. The putative protein sequence of this gene was then utilized to detect *DHN* genes from the published *V. vinifera* cv. Pinot Noir clone PN40024 genome sequence [30] via BLAST analysis. Four *DHN* genes were identified, all of which contained a K-segment. These genes were designated *VvDHN1* (corresponding in sequence to the previously identified *V. vinifera DHN1a*) [GenBank:XM_03631828], *VvDHN2* [GenBank:XM_002285883], *VvDHN3* [GenBank:CAN73166], and *VvDHN4* [GenBank:XM_002283569].

The three remaining *DHN* genes were cloned from *V. yeshanensis* acc. Yanshan-1 seed-specific cDNA using primers derived from the *V. vinifera* sequences and were designated *VyDHN2* [GenBank:JQ408442], *VyDHN3* [GenBank:JQ408443], and *VyDHN4* [GenBank:JQ408444]. While only 25 amplification cycles were required to clone both *VyDHN2* and *VyDHN4*, 40 cycles were required in the case of *VyDHN3*. Subsequently, all four *V. yeshanensis* genes were amplified from genomic DNA to identify intronic regions [GenBank:JF896520, JF896556, JF896557, and JF896558, respectively]. In both species, all four *DHN* genes consisted of two exons separated by one intron present within the S-segment.

**Figure 1:**
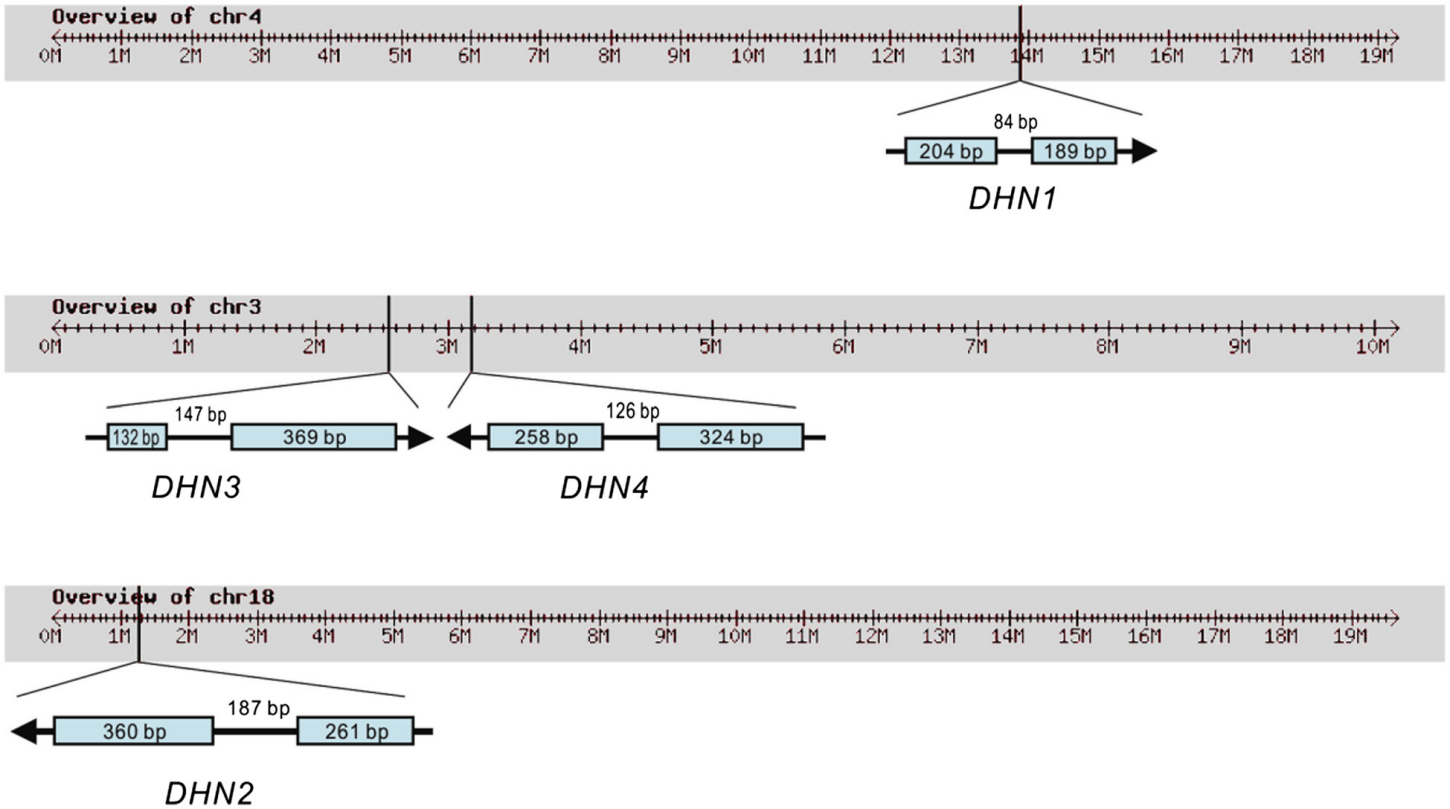
**Predicted structure and chromosomal localization of* V. vinifera DHN *genes**. Chromosomal localization of the four *DHN* genes was determined from the *V. vinifera* cv. Pinot Noir clone PN40024 genome sequence. Exons are represented as gray boxes, with respective lengths indicated within each box. Lengths of introns, which are denoted by a line between exons, are indicated above each line. Arrows indicate direction of transcription in each case.

In terms of nucleotide similarities, virtually no sequence identity was detected between the four *DHN* coding sequences. However, high levels of homology were noted between matching genes belonging to the two different species, with 97% (*DHN4*) and 99% (*DHN1*, *DHN2* and *DHN3*, respectively) identity at the nucleotide level. In terms of chromosomal localization, while *VvDHN1* and *VvDHN2* were located on chromosomes 4 and 18, respectively, *VvDHN3* and *VvDHN4* were mapped to chromosome 3 in opposite orientations (Figure 1).

### Characterization and comparison of deduced DHN proteins

**Figure 2:**
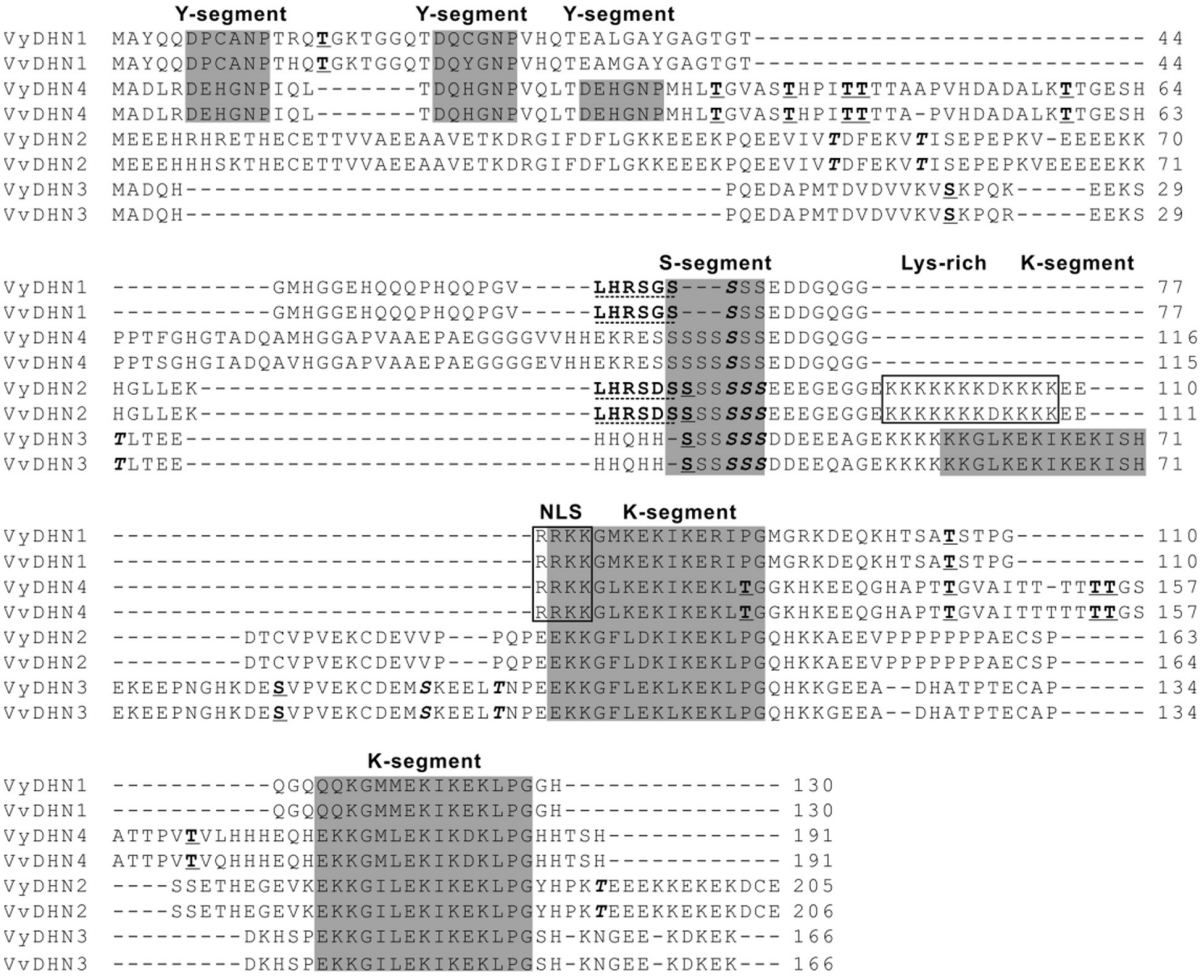
**Sequence alignment of DHN proteins from* V. yeshanensis *and* V. vinifera *.** Y-segments, S-segments and K-segments are denoted by gray shading. NLS and Lys-rich segments are framed by a black line. Phosphorylation sites are in bold, with PKC sites underlined with a single line, CK2 sites in italics, and SnRK-10 sites underlined with a dotted line.

Protein sequences were deduced from the corresponding *V. vinifera* and *V. yeshanensis DHN* cDNA sequences, and were composed of 130–206 amino acids exhibiting 97-99% identity at the amino acid level between the two species. Both K- and S-segments were found to be highly conserved between members of the *V. vinifera* and *V. yeshanensis* DHN families, whereas remaining regions displayed relatively low amino acid identity between the four genes. Furthermore, while NLS domains were identified in both DHN1 and DHN4 proteins, a Lys-rich segment was only present in DHN2 (Figure 2). Based on the presence and number of K-, S- and Y-motifs (Figure 2), the four DHNs from each species were classified as either Y_2_SK_2_- (DHN1), SK_2_- (DHN2), SK_3_- (DHN3), or Y_3_SK_2_-type (DHN4) proteins (Figure 2; Table 1).

**Table 1:** **Characteristics of DHN proteins in*V. yeshanensis*and*V. vinifera***

**Name**	**Type**	**No. of Residues**	**MW (kDa)**	**pI**	**GRAVY**	**PKC No**	**CK2 No**	**SnRK2 No**	**Expression**
*VyDHN1*	Y_2_SK_2_	130	13.9	9.36	−1.425	2	1	1	Stress + Seed
*VvDHN1*	Y_2_SK_2_	130	13.9	9.27	−1.459	2	1	1	
*VyDHN2*	SK_2_	205	23.4	5.21	−1.527	1	6	1	Constitutive + Stress + Seed
*VvDHN2*	SK_2_	206	23.5	5.20	−1.514	1	6	1	
*VyDHN3*	SK_3_	166	18.8	5.81	−1.736	3	6	0	Seed (weak)
*VvDHN3*	SK_3_	166	18.9	5.92	−1.739	3	6	0	
*VyDHN4*	Y_3_SK_2_	191	20.1	6.35	−0.959	10	1	0	Seed
*VvDHN4*	Y_3_SK_2_	191	20.1	6.26	−1.030	10	1	0	

MW (molecular weight), pI (isoelectric point) and GRAVY (grand average of hydropathy) were predicted based on amino acid composition. PKC, CK2 and SnRK2 refer to specific phosphorylation sites. Expression information is based on the RT-PCR experiments described in Figures 4 through 7.

All members of the DHN family in the two grapevine species analyzed were found to be highly hydrophilic, with GRAVY values ranging from −0.959 to −1.527 and theoretical pIs from 5.20 to 9.36 (Table 1). DHN1 and DHN4, which were Y_n_SK_n_-type DHNs, possessed a higher pI than the SK_n_-type DHNs (DHN2 and DHN3) in both species. In terms of acidity, our analyses indicated that DHN1 was the sole basic protein, while DHN2 was the most acidic.

Many phosphorylation sites were also predicted within each of the DHN protein sequences analyzed, with DHN1 and DHN4 containing a higher number of putative protein kinase C (PKC) phosphorylation sites than casein kinase 2 (CK2) phosphorylation sites, and DHN2 and DHN3 containing a higher number of CK2 sites than PKC sites (Table 1; Figure 2). In addition, a recently identified conserved motif (LXRXXS) phosphorylated by an Snf1-related kinase (SnRK2-10) [31] was identified in both DHN1 and DHN2 proteins.

While all four grapevine DHNs were found to be rich in disordered regions and contained relatively few helix or strand motifs (see Additional file 2), the Y_n_SK_n_-type proteins (DHN1 and DHN4) displayed the highest disorder index and least helix/strand-motifs. Additionally, of the few helix motifs identified, most were located within K-segments, which is consistent with the findings of a previous study of DHN protein structure [14].

### Phylogenetic analysis of *V. vinifera* and *V. yeshanensis* DHNs

**Figure 3:**
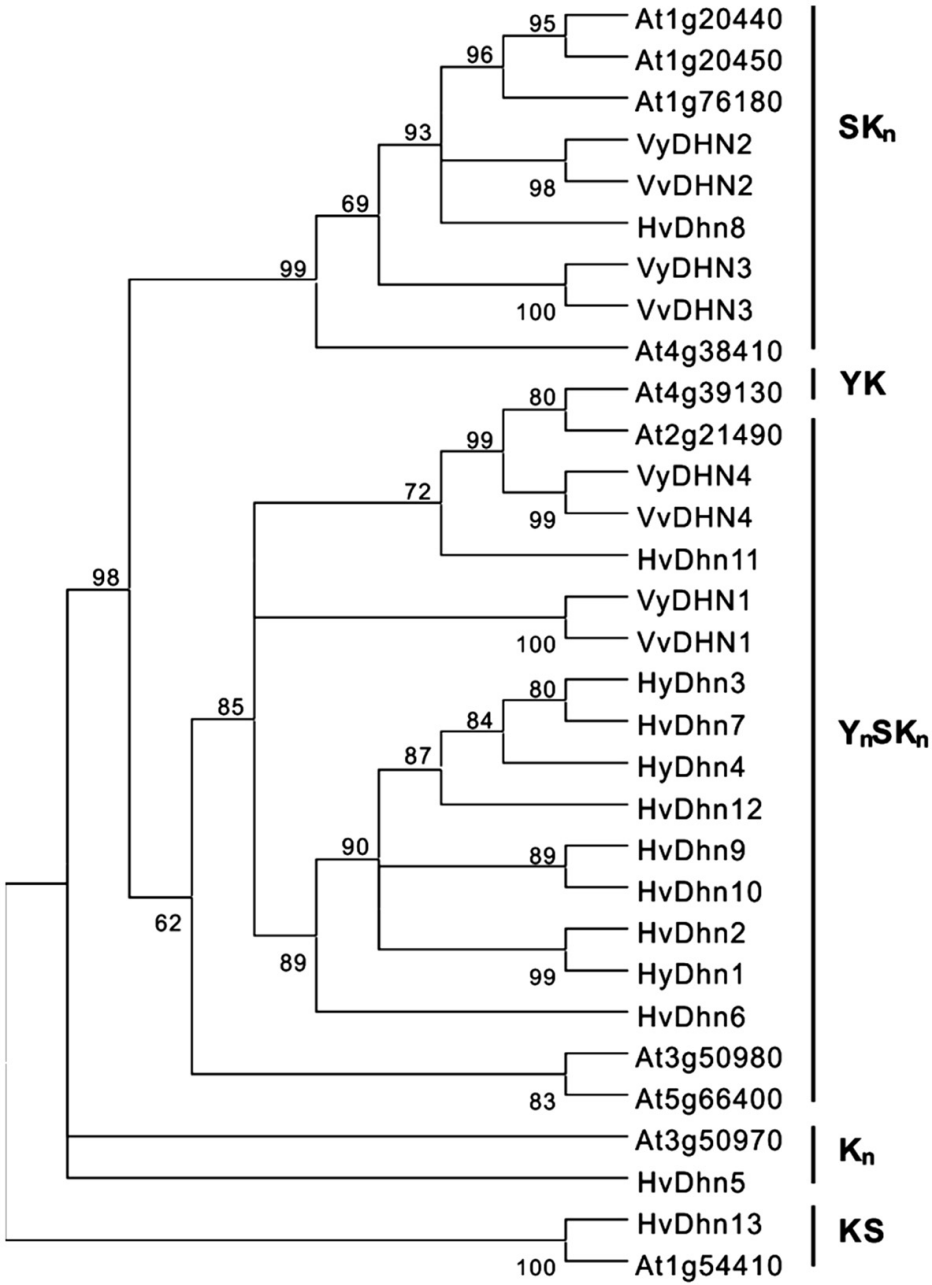
**Phylogenetic relationships between grapevine, barley and Arabidopsis DHN proteins.** The unrooted dendrogram was constructed with the PhyML tool using the maximum likelihood method based on a complete protein sequence alignment of DHNs from *Arabidopsis thaliana* (At), *Hordeum vulgare* (Hv), *V. vinifera* (Vv), and *V. yeshanensis* (Vy). The bootstrap value is given for each node. GenBank accession numbers are as follows: VvDHN1 [XM_03631828], VvDHN2 [XM_002285883], VvDHN3 [CAN73166], VvDHN4 [XM_002283569], VyDHN1 [JF900497], VyDHN2 [JQ408442], VyDHN3 [JQ408443], VyDHN4 [JQ408444], At1g20440 [AY114699], At1g20450 [AF360351], At1g54410 [NM_104319], At1g76180 [AF339722], At2g21490 [BT000900], At3g50970 [NM_114957], At3g50980 [NM_114958], At4g38410 [NM_120003], At4g39130 [NM_120073], At5g66400 [AY093779], HvDhn1 [AF043087], HvDhn2 [AF181452], HvDhn3 [AF181453], HvDhn4 [AF181454], HvDhn5 [AF181455], HvDhn6 [AF181456], HvDhn7 [AF181457], HvDhn8 [AF181458], HvDhn9 [AF181459], HvDhn10 [AF181460], HvDhn11 [AF043086], HvDhn12 [AF155129], HvDhn13 [AY681974].

To date, DHN families from both barley and Arabidopsis have been thoroughly characterized at the genomic level [18,32-35]. Therefore, to provide a further understanding of the relationships between the *V. vinifera* and *V. yeshanensis* DHNs, we conducted a phylogenetic analysis of these genes via comparison with those from barley and Arabidopsis. Overall, we found the number of *DHN* genes in the grapevine species (four) to be smaller than that in either barley (thirteen) or Arabidopsis (ten). Based on our phylogenetic results, the DHNs could be divided into four groups, corresponding to Y_n_SK_n_-, SK_n_-, K_n_-, and KS-type proteins (Figure 3), where the Arabidopsis YK-type DHN (At4g39130) was included within the Y_n_SK_n_ group. As expected from our classification of the grapevine DHN sequences based on the presence of various conserved segments, the grapevine DHN1 and DHN4 proteins were grouped together with the Y_n_SK_n_-type DHNs of Arabidopsis and barley, while the grapevine DHN2 and DHN3 proteins were grouped with the SK_n_-type DHNs of Arabidopsis and barley. Interestingly, both grapevine species lacked KS- and K_n_-type DHNs; groups which are present in both barley and Arabidopsis. 

### Expression profiles of *DHN* transcripts in various tissues and developmental stages

**Figure 4:**
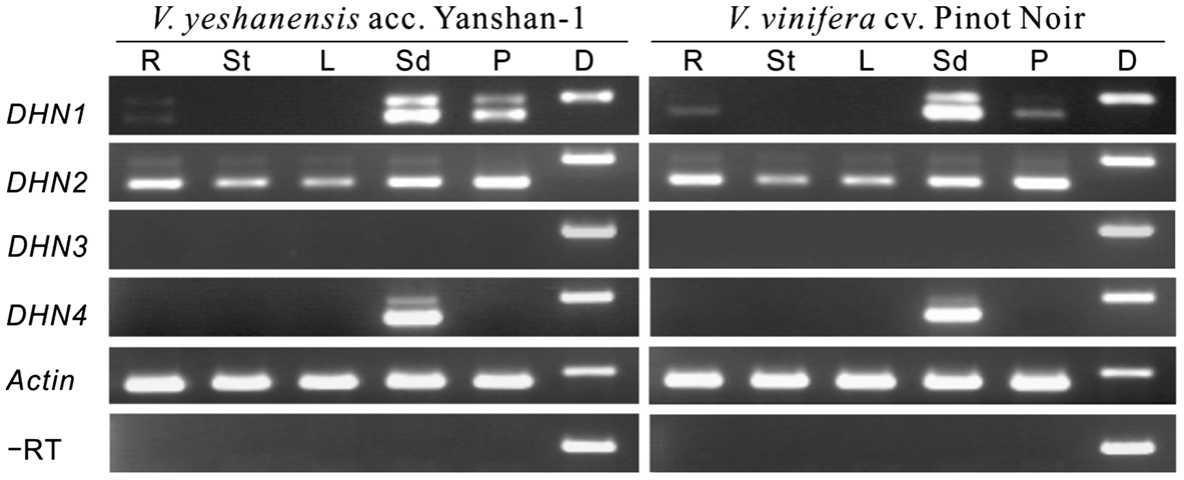
**Expression of* DHNs *in various organs of* V. yeshanensis *and*V. vinifera.*** Total RNA was isolated from root (R), stem (St), leaf (L), seed (Sd) and fruit peel (P) at veraison, and was quantified using a Nanodrop spectrophotometer (Nanodrop Products, Wilmington, DE, USA). 1 μg DNase-treated total RNA was used as template for first-strand cDNA synthesis in a final volume of 20 μl, and subsequently 1 μl of this reaction was utilized for PCR amplification in a volume of 25 μl. Genomic DNA (D) was utilized as the positive control. RNA without reverse transcriptase was used as the negative control. The grapevine *actin1* fragment was amplified as an internal control. 15 μl of PCR products were separated on a 1.5 % agarose gel containing ethidium bromide in each case.

To elucidate the physiological functions of different members of the *DHN* family in *V. vinifera* and *V. yeshanensis*, the expression of *DHN* genes was investigated at veraison in roots, stems, leaves, seeds, and fruit peels using semi-quantitative RT-PCR (Figure 4). Both species exhibited highly similar expression profiles for their matching *DHN* genes. Results suggested that under normal growth conditions, *DHN1* was mainly expressed in seeds, with very low levels present in the roots. *DHN2* was constitutively expressed in all tissues; however, weaker levels of expression were noted in leaves and stems than in the other organs tested. *DHN3* was undetectable under the current experimental conditions, suggesting that it was not expressed, or was at levels too low to be detected using this method, in these tissues. The *DHN4* genes, on the other hand, were expressed specifically in seeds. These results indicate that the four *DHN* genes that make up the *V. vinifera* and *V. yeshanensis DHN* gene families, respectively, exhibited very distinct expression patterns in the organs tested.

Intriguingly, reactions in which *DHN1*, *DHN2* and *DHN4* were amplified often exhibited two bands: one that corresponded in size to a spliced transcript, and one that seemed to be closer in size to the band amplified from genomic DNA (ie. containing an unspliced intron). The fact that no amplification products were obtained in negative control reactions lacking reverse transcriptase confirmed that template RNA was free from contaminating genomic DNA. Therefore, it seems that these larger transcript variants resulted from the presence of unspliced *DHN* transcript variants (either mRNA or pre-mRNA) within the total RNA pool.

**Figure 5:**
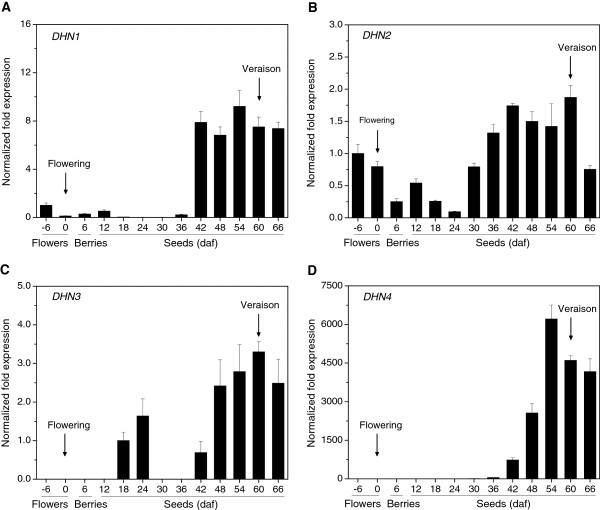
**Expression of* DHN *s in* V. vinifera *during seed development.** Total RNA was isolated from floral buds harvested 6 days prior to flower opening (−6 daf), flowers were harvested on the day of flower opening (0 daf), young berries were harvested 6 daf, and seeds were harvested from 12–66 daf. Transcript levels of *DHN1*, *DHN2*, *DHN3*, *DHN4* normalized to the levels of the internal control, *actin1*, were determined using real-time qRT-PCR analysis. Each block represents the mean relative fold-change compared to baseline levels of expression from three experiments, while bars indicate standard deviations. In the case of *DHN1*, *DHN2* and *DHN4*, baseline expression levels were those measured at −6 daf. In the case of *DHN3*, baseline expression was set to that measured at 18 daf as this was the first time point at which expression was noted. Times indicating flowering and veraison are indicated by arrows.

To gain a more precise understanding of grapevine *DHN* expression during the process of seed development, inflorescences and berries of *V. vinifera* were harvested from flowering to veraison and used for qRT-PCR expression analyses. *DHN1* and *DHN2* were found to be expressed in floral buds even 6 d before opening of the flowers (Figure 5A and B), after which time their transcripts decreased during the middle stages of embryogenesis, and were strongly up-regulated once again during later stages of embryogenesis. Low levels of *DHN3* were detected in both mid- and late-stages of seed development (Figure 5C) while *DHN4* transcripts were only detectable during late stages of embryogenesis, with peak expression developing just prior to veraison (Figure 5D).

### Response of *DHN* gene expression to various abiotic and biotic stresses

**Figure 6:**
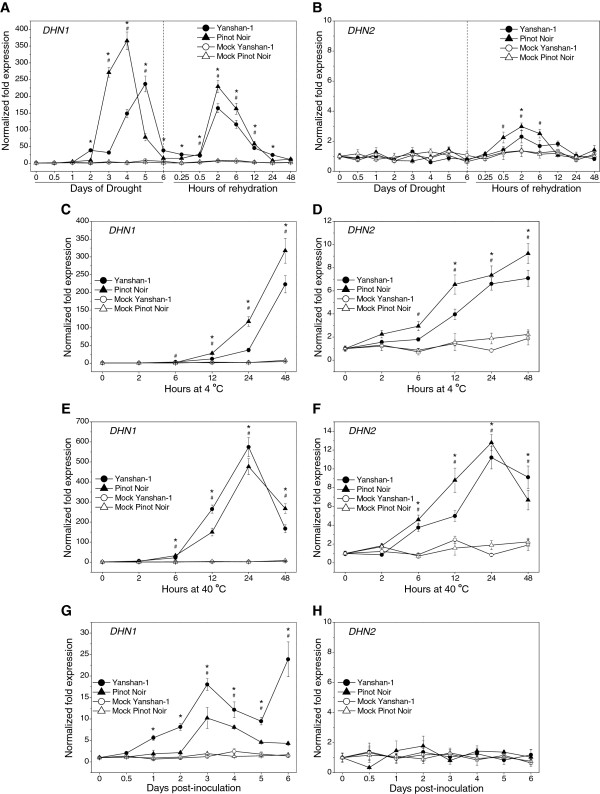
**Expression profiles of grapevine* DHN1 *and* DHN2 *under abiotic and biotic stress.** Total RNA was extracted from the leaves of *V. yeshanensis* and *V. vinifera* treated with drought-rehydration (**A**, **B**), 4 °C (**C**, **D**), 40 °C (**E**, **F**), and inoculation with *E. necator* (**G**, **H**). Samples were taken at the indicated times, with time zero samples harvested immediately prior to treatment. Normalized transcript levels of *DHN1* and *DHN2* were determined by real-time qRT-PCR analysis, with the *actin1* gene serving as an internal control. Each point represents the mean relative fold-change compared to baseline levels of expression from three experiments, while bars indicate standard deviations. Baseline expression levels were those measured just prior to treatment at time = 0. Asterisks (*) and number signs (#) indicate significant increases (p < 0.05) in expression levels of target transcripts from *V. yeshanensis* and *V. vinifera*, respectively, compared to the mock-treated controls.

In an attempt to determine whether *DHN1*, *DHN2*, *DHN3* and *DHN4* exhibited stress-responsiveness, we analyzed the expression levels of all four genes in the leaves of three *V. vinifera* and *V. yeshanensis* plants, respectively, that had been subjected to various stress conditions using real-time qRT-PCR. Neither *DHN3* nor *DHN4* from either species exhibited detectable levels of expression under any of the conditions tested here, including drought, cold, heat, or *E. necator* infection; therefore, only *DHN1* and *DHN2* will be discussed further in this section. In the case of drought conditions, although we found that *DHN1* was induced by this treatment in both species (Figure 6A), we noted slight differences between the two species. While *DHN1* transcripts increased in *V. yeshanensis* between 1–2 d after the drought treatment began and reached a maximum induction of a 237-fold increase compared to baseline expression levels 5 d after treatment, its homologue in *V. vinifera* was delayed in its exhibition of a response (between 2–3 d) and reached a higher maximum induction of 366-fold compared to baseline levels*.* Conversely, *DHN2* did not appear to respond to drought treatment in either of the two species tested (Figure 6B). Interestingly, transcripts of both *DHN1* and *DHN2* also exhibited up-regulation in both grapevine species shortly after rehydration (Figure 6 A and B), with a maximum level of expression achieved after approximately 2 h.

Following cold and heat treatment, both *DHN1* and *DHN2* were induced in *V. vinifera* and *V. yeshanensis*. While *DHN2* transcripts increased gradually between 0 h and 48 h following initiation of cold stress in both species, *DHN1* transcripts exhibited a more sudden onset of up-regulation in response to cold beginning between 6–12 h after treatment (Figure 6 C and D). In the case of heat stress, both *DHN1* and *DHN2* transcripts increased to maximum levels 24 hours following the initiation of treatment in both species, and then subsequently decreased (Figure 6 E and F).

To determine whether grapevine *DHNs* were responsive to biotic stress, the levels of *DHN* gene expression were tested in the leaves of *V. vinifera* and *V. yeshanensis* inoculated with *E. necator*, which is the causative agent of grapevine powdery mildew. Results suggested that only *DHN1* was induced by *E. necator*, whereby transcripts increased gradually to a maximum at 3 d post-inoculation (dpi) and then decreased slowly after this point in both species. Interestingly, *V. yeshanensis* exhibited a higher peak level of expression (18-fold increase compared to expression levels immediately prior to treatment) than *V. vinifera* (10-fold increase), as well as an additional sharp increase in *DHN1* expression 6 dpi (Figure 6G). In contrast, *DHN2* did not appear to respond to *E. necator* inoculation in either grapevine species (Figure 6H).

### Response of *DHN* gene expression to levels of various signaling molecules

**Figure 7:**
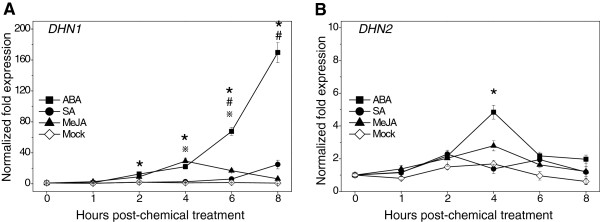
**Expression profiles of grapevine* DHN1 *and* DHN2 *following treatment with various hormones.** Total RNA was extracted from the leaves of *V. vinifera* sprayed with 50 μM MeJA, 100 μM SA and 100 μM ABA (**A**, **B**). Samples were taken at the indicated times, with time zero samples harvested immediately prior to spraying. Normalized transcript levels of *DHN1* and *DHN2* were determined by real-time qRT-PCR analysis, with the *actin1* gene serving as an internal control. Each point represents the mean relative fold-change compared to baseline levels of expression from three experiments, while bars indicate standard deviations. Baseline expression levels were those measured just prior to treatment at time = 0. Asterisks (*), number signs (#) and reference marks (※) indicate significant differences (p < 0.05) in expression levels of target transcripts from *V. vinifera* treated with ABA, SA and MeJA, respectively, compared to the mock-treated controls.

The responses of plants to abiotic and biotic stress are generally mediated by abscisic acid (ABA) and salicylic acid (SA)/jasmonic acid (JA), respectively. To determine whether the induction of grapevine *DHNs* under stress conditions was related to any of these signaling molecules, *DHN* expression was investigated in leaves from three *V. vinifera* plants treated with ABA, SA or MeJA, respectively. As was the case for studies involving abiotic and biotic stress treatment, no detectable levels of *DHN3* or *DHN4* expression could be detected in leaves treated with any of these chemicals. While *DHN1* was induced by ABA, SA and MeJA (Figure 7A), the most considerable up-regulation (160-fold compared to baseline levels) was noted 8 h following application of ABA. *DHN1* transcripts from leaves treated with MeJA reached a maximum induction of ~20-fold compared to baseline levels approximately 4 h following application, whereas a similar level of induction was reached 8 h following treatment with SA. In the case of *DHN2* transcripts, expression reached a maximum 5-fold induction compared to baseline levels 4 h after application of ABA. Although slight modifications were also noted in *DHN2* expression levels following SA and MeJA applications, respectively, they differed only slightly from changes observed in untreated samples (Figure 7B).

### Comparison of *cis*-regulatory elements in the upstream regulatory regions of grapevine *DHN* genes

All four *DHN* promoters (including 1500-bp of sequence upstream of the translational start codon) were cloned from *V. yeshanensis* acc. Yanshan-1 [GenBank: JF899925 for *VyDHN1*, GenBank: JX110839 for *VyDHN2*, GenBank: JX110840 for *VyDHN3*, and GenBank: JX110841 for *VyDHN4*] using primers derived from the corresponding *V. vinifera* sequences. High levels of homology were detected between matching promoters from the two different species, with 94% (*DHN1*), 96% (*DHN2*), and 92% (*DHN4*) identity at the nucleotide level. The promoter of *DHN3*, on the other hand, only exhibited 84% identity between the two species due to a 205-bp deletion in this sequence from *V. yeshanensis*.

To elucidate whether the differential expression patterns of the four grapevine *DHN* genes correlate with transcriptional regulation via their promoters, upstream regions of each gene from *V. yeshanensis* and *V. vinifera* were scanned for putative c*is*-regulatory elements using the PlantCARE database [36]. Nucleotide sequences including 1500-bp upstream of each start codon were obtained from the *V. vinifera* cv. Pinot Noir clone PN40024 genome database [30] , while those from *V. yeshanensis* were obtained directly via cloning, and *cis*-regulatory elements were classified into three groups according to their potential responsive functions: abiotic stress-related elements, biotic stress-related elements, and seed development-related elements. Abiotic stress-related elements comprised ABA-responsive elements (ABRE), dehydration-responsive elements (DRE), heat shock-responsive elements (HSE), and low temperature-responsive elements (LTR). Biotic stress-related elements included MeJA-responsive elements (MeJA-RE), salicylic acid-responsive elements (TCA), as well as stress- and defense-responsive elements (TC-rich repeats). Seed development-related elements comprised only endosperm-specific expression elements (Skn-1 motif).

**Figure 8:**
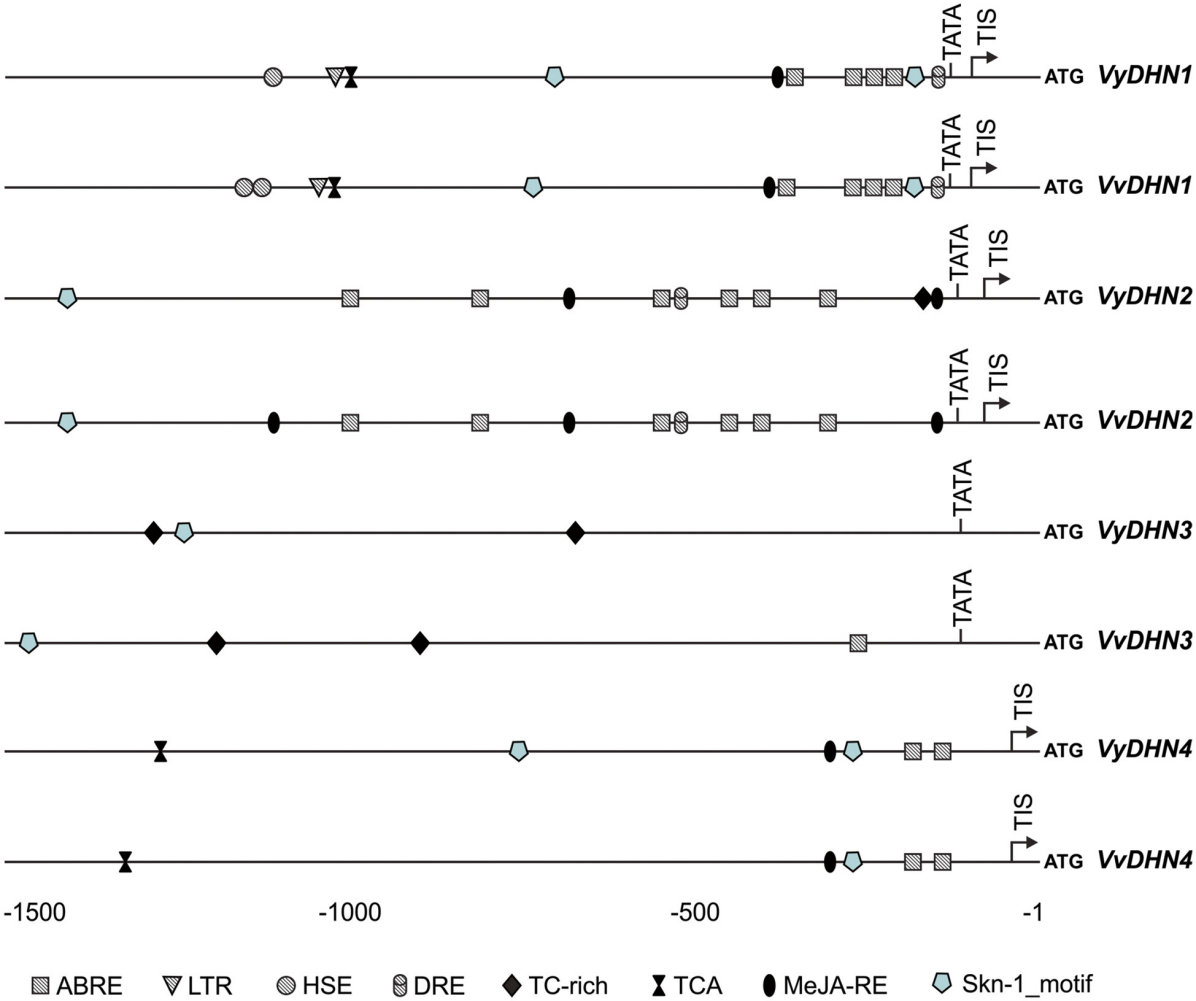
**Location of putative regulatory elements in the promoter regions of the* V. yeshanensis *and* V. vinifera DHN *genes**. Promoter regions comprising 1500-bp of sequence upstream from the translational start sites were obtained from the published *V. vinifera* cv. Pinot Noir clone PN40024 genome sequence. In addition, the matching promoter regions were also cloned from *V. yeshanensis* acc. Yanshan-1 genomic DNA. Putative *cis*-regulatory elements were predicted using the PlantCARE website and those involved in stress-induction and seed development were mapped. Recognition sequences are shown in Table 2.

All *cis*-regulatory elements found in the *V. yeshanensis* and the *V. vinifera DHN* promoters, which exhibited a similar composition and distribution of putative regulatory elements between corresponding promoters, are shown in Table 2. There were obvious differences in the abundance and distribution of *cis*-regulatory elements in the four *DHN* promoters analyzed (Figure 8). The *VyDHN1* and *VvDHN1* promoters had the most diverse collection of putative *cis*-regulatory elements, including several involved in stress-response, seed development, and hormone signaling (Table 2). In the case of the *VyDHN2* and *VvDHN2* promoters, HSE, LTR and TCA elements were lacking when compared to *VyDHN1* and *VvDHN1*. Only a small number of potential *cis*-elements were identified in the *VyDHN3*, *VvDHN3*, *VyDHN4* and *VvDHN4* promoters. In the *VvDHN3* promoter, an ABRE, Skn-1 and two TC-rich elements were scattered evenly throughout the promoter, while the ABRE element was absent in that of *VyDHN3*. In *VvDHN4*, two ABREs, a Skn-1 and a MeJA-RE were concentrated in the 3’ region of the promoter, while a TCA element was present in the 5’ region. A similar distribution was noted in the *VyDHN4* promoter, with an additional Skn-1 motif present 765-bp upstream of the translational start codon.

**Table 2:** **Regulatory elements involved in stress-, pathogen- and embryogenesis-responsiveness in grapevine*DHN*promoter regions**

***Cis*-element**	**Sequence**	Number of*cis*-elements^*^				**Function**	**References**
		*DHN1*	*DHN2*	*DHN3*	*DHN4*		
ABRE	CACGTG	4/4	6/6	0/1	2/2	Abscisic acid responsiveness	[37]
DRE	ACCGAC	1/1	1/1	0/0	0/0	Drought and cold responsiveness	[38]
HSE	AGAAAATTCG	1/2	0/0	0/0	0/0	Heat stress responsiveness	[39]
LTR	CCGAAA	1/1	0/0	0/0	0/0	Low-temperature responsiveness	[40]
TC-rich repeats	ATTTTCTTCA	0/0	0/0	2/2	0/0	Stress and defense responsiveness	[41]
MeJA-RE	CGTCA	1/1	2/3	0/0	1/1	MeJA-responsiveness	[42]
TCA element	CCATCTTTTT	1/1	1/0	0/0	1/1	Salicylic acid responsiveness	[43]
Skn-1_motif	GTCAT	2/2	1/1	1/1	2/1	Endosperm expression	[44]

^*^ The first number indicates the number of *cis*-regulatory elements within the *V. yeshanensis DHN* promoter while the second number indicates the number of *cis*-regulatory elements within the *V. vinifera DHN* promoter.

## Discussion

Dehydrins are believed to play a fundamental role in the response of plants to various abiotic and biotic stresses. They make up a multigene family with 10 members in Arabidopsis [18,35], 8 members in rice [45], 13 members in barley [23], and 11 members in poplar [46]. However, only 4 *DHN* genes were identified in the published *V. vinifera* genome sequence [30,47], including two Y_n_SK_n_-type DHNs (*DHN1* and *DHN4*) and two SK_n_-type DHNs (*DHN2* and *DHN3*). Neither K_n_- nor KS-type DHNs were found in this species, which differs from the *DHN* gene families from other plant species characterized to date and suggests that these types of genes may have been lost in grape species (Figure 2).

Expansion of the *DHN* family has generally occurred through tandem duplication events and whole-genome duplications. For example, At1g20440/At1g20450, At3g50970/At3g50980, and At4g38140/at4g39130 arose from tandem duplications, while At1g20450/At1g76180, At2g21490/At4g39130, and At3g50970/At5g66400 arose from a whole-genome duplication event in Arabidopsis [18], which together resulted in an increase of 6 *DHN* genes. Similarly, whole-genome and tandem duplication events were responsible for an increase of 3 and 2 *DHN* genes, respectively, in poplar [46]. At least 3 *DHN* genes arose from tandem duplication events in rice [45], and it is possible that the two clusters of *DHN* genes on chromosomes 5 H and 6 H in *H. vulgare*, respectively [32], which show a high level of sequence identity within each cluster, may have resulted from tandem duplication events.

While the genomes of poplar, rice and Arabidopsis have undergone at least one recent whole-genome duplication event, the grapevine genome has not [30]. Instead, the four grapevine *DHNs* have likely arisen from an ancestral genome [30], which is consistent with the low level of sequence similarity between the four sequences. However, *DHN3* and *DHN4* lay in close proximity on chromosome 3 in *V. vinifera*, which implies that one of them may have arisen through a tandem duplication event despite their low level of identity. Therefore, it seems that the relatively low number of *DHN* genes in grapevine may simply be due to a lack of duplication events in this genus. Indeed, it has been suggested that gene family expansion in grapevine has been selective, occurring mainly in those genes involved in aromatic features [30].

*In silico* characterization of *V. vinifera* and *V. yeshanensis* DHN protein sequences suggested they were all highly hydrophilic and disordered, but with distinct differences in pI, kinase specificity and content of functional motifs. The two Y_n_SK_n_-type DHNs (DHN1 and DHN4) possessed a higher pI than the SK_n_-type DHNs (DHN2 and DHN3). Since positively charged DHN proteins bind negatively charged membranes with a high affinity [13], it follows that the Y_n_SK_n_-type DHNs, and especially DHN1, could very well bind with the cell membrane in grapevine. It has been suggested that the binding of DHNs to membranes may be modulated by phosphorylation through an alteration of net charge [22]. The DHN1 and DHN4 proteins from grapevine were found to contain a higher number of putative PKC sites than CK2 sites, whereas DHN2 and DHN3 bore a higher number of putative CK2 sites than PKC sites (Table 1; Figure 2). This finding is in agreement with a previous suggestion that Y_n_SK_n_-type DHNs are mainly phosphorylated by PKC, while SK_n_-type DHNs are mainly phosphorylated by CK2 [22].

DHN proteins with similar physicochemical properties often also exhibit similar expression patterns. For example, while genes encoding alkaline Y_n_SK_n_-type DHNs, such as *At5g66400HvDhn1HvDhn2HvDhn3HvDhn4HvDhn7HvDhn9* and *HvDhn10*, are generally induced by both embryogenesis and various types of stress [18,23], those encoding acidic SK_n_- and K_n_S-type DHNs, such as *At1g20440At1g20450At1g76180At1g5410HvDhn8* and *HvDhn13*, are expressed constitutively in vegetative tissues and are also up-regulated by various types of stress [18,23,35]. The expression patterns of grapevine *DHN1* and *DHN2* agree with those predicted by their classification, which suggests that this holds true in the species analyzed here.

Even though the grapevine DHN1 and DHN4 (Y_n_SK_n_-type), as well as DHN2 and DHN3 (SK_n_-type), proteins are grouped into only two classes, all four members of the grapevine *DHN* family exhibited very distinct patterns of expression (Figure 4, Figure 5, Figure 6, and Figure 7). We found grapevine *DHN1* to be induced by drought, cold, heat, *E. necator*, and to be expressed during late stages of embryogenesis, which corresponds well with previous reports [28,29]. Conversely, *DHN2* was found to be constitutively expressed in vegetative tissues and was up-regulated under cold and heat conditions, as well as during late embryogenesis (Figure 4, Figure 5, Figure 6, and Figure 7). In contrast, very low levels of *DHN3* expression were only detected during seed development with no induction observed in vegetative tissues following any of the stress or signaling molecule treatments studied here. Correspondingly, although no *DHN3* transcripts could be identified in GenBank’s EST database, a large number (tens to hundreds) of the remaining grapevine *DHN* genes were (data not shown), which suggests that *DHN3* is expressed at undetectable levels in most tissue types. Likewise, *DHN4* was also specifically expressed during late embryogenesis, but at far higher levels than *DHN3* (Figure 4 and Figure 5). These results suggest that the function of the grapevine *DHN* genes is likely divergent, but may also exhibit some level of overlap.

The accumulation of *DHN*s in plants is believed to have been associated with the acquisition of desiccation tolerance in these organisms [12] and expression levels of these genes in vegetative tissues has generally been found to be higher in drought-tolerant cultivars than in their susceptible counterparts [48-51]. However, this is not always the case, as differences in expression levels between tolerant and sensitive genotypes are often dependent on the type of *DHN*, as well as the duration of the stress. While both *V. yeshanensis* and *V. vinifera* have been found to exhibit some tolerance to drought, the former exhibits a higher tolerance than the latter and also displays resistance to cold [25,26]. In the case of induction via temperature stress, both *DHN1* and *DHN2* exhibited cold and heat responsiveness; however, *DHN1* appeared to be far more responsive than *DHN2* (Figure 6 C-F). Interestingly, induction tended to be higher in *V. vinifera* than *V. yeshanensis*, which is contrary to the levels of temperature sensitivity in these two species.

Conversely, among the four grapevine *DHN* genes tested, only *DHN1* was induced by drought stress in vegetative tissues. This gene was up-regulated between 1–2 d after the initiation of drought conditions in *V. yeshanensis*, while its expression level at this time remained unchanged in *V. vinifera*, suggesting that the expression of *DHN1* was quicker to respond to drought in the tolerant genotype. However, *V. yeshanensis* did not show a higher level of *DHN1* expression than *V. vinifera* at 3 and 4 d following treatment (Figure 6 A and B). A similar situation has been observed in barley, where the *HvDhn6* gene was expressed earlier in tolerant cultivars than susceptible cultivars under drought conditions, but at lower levels than the susceptible cultivars at time points that were further from the commencement of drought conditions [49,50].

Generally, *DHN* genes are up-regulated under drought stress and down-regulated following rehydration [52-55]. However, in this study, the grapevine *DHN1* and *DHN2* genes also displayed induction 2 h post-rehydration (Figure 6 A and B). In line with this, it has been found previously that leaf ABA content increases during early phases following re-hydration [56]. Therefore, the up-regulation of grapevine *DHNs* after rehydration may correspond to a change in leaf ABA content, since both genes were found to be responsive to this plant hormone (Figure 7 A and B).

Recent studies have indicated that *DHN*s are also responsive to pathogen infection. For example, a *DHN* gene can be utilized to predict blast resistance in rice [57], and the Arabidopsis *LTI30* and *RAB18* genes have been found to be up-regulated by inoculation with powdery mildew [18]. This pathogen-induced expression of *DHNs* may provide another important function for this type of gene in disease resistance. In the current study, only *DHN1* was found to be up-regulated in *V. yeshanensis* and *V. vinifera* following inoculation with *E. necator*, which is the causative agent of grapevine powdery mildew (Figure 6G). Intriguingly, the expression level of *DHN1* was higher in the resistant *V. yeshanensis* than in the susceptible *V. vinifera*, and a second induction event was also apparent in *V. yeshanensis* that was lacking in *V. vinifera*. These results suggest that *DHN1* may participate in powdery mildew resistance in *V. yeshanensis*.

*DHN1* from *V. vinifera* was also induced by the signaling molecules SA and MeJA, which are known to be involved in defense response, providing further evidence that it could play a role in systemic acquired resistance [58]. It has been demonstrated previously that a number of pathogen-responsive genes were up-regulated in transgenic Arabidopsis plants overexpressing *DHN-5*, which implies that DHNs might act as stress signaling molecules that regulate defense genes [11]. This may also be the case for the *DHN1* genes from grapevine (Figure 7A), although further experiments will be necessary to show this definitively.

The expression of stress-responsive genes depends upon the presence of *cis*-regulatory elements in their promoter regions [59], as has been shown to be the case for barley *DHN* genes [32]. The four grapevine *DHN* promoters exhibited distinct differences in the composition and distribution of putative regulatory elements held within them. ABREs, which are one of the most common *cis*-elements in the *DHN* promoters, likely played a role in the induction of *DHN1* by ABA, mediating its expression under drought conditions. Indeed, when taken together, all of the putative regulatory elements identified within both the *DHN1* and *DHN2* promoters could account for their up-regulation by a variety of stresses and their corresponding signal molecules (Figure 8). In contrast, relatively few regulatory elements were found in the *DHN3* and *DHN4* promoters, which corresponds with the fact that neither of these genes were found to be induced by any of the stresses or signaling molecules analyzed.

The quantity and location of regulatory elements could also have a profound effect on grapevine *DHN* expression. It has been found previously that a single copy of an ABRE is not sufficient for ABA-responsive induction of transcription [59]. In this study, a higher number of ABRE elements were located in *DHN1* and *DHN2* promoters than in *DHN3* and *DHN4* promoters (Figure 8); correspondingly, the two former genes were responsive to induction by ABA, whereas *DHN3* and *DHN4* were not. Furthermore, the Skn-1 motif, which has been shown previously to confer a promoter with endosperm-specific expression [60], was found in all four grapevine *DHN* promoters. However, these motifs were located much nearer to the translational start codon in *DHN1* and *DHN4* promoters than in *DHN2* and *DHN3* promoters, which may provide an explanation for increased up-regulation of *DHN1* and *DHN4* during late embryogenesis (Figure 4 and Figure 5).

## Conclusions

The *DHN* gene family was identified in a genome-wide search of the published genome sequence of *V. vinifera*, and the corresponding homologues were isolated from *V. yeshanensis*. A large expansion of the *DHN* family apparently did not take place in grapevine, although it has been a common occurrence in other plants. The four grapevine *DHN* genes shared a low sequence identity, and exhibited clear differences in physicochemical properties and expression profiles, which indicates functional diversification within the grapevine *DHN* family. *DHN1* appeared to be the principal stress-responsive gene in grapevine species, and was induced not only by various abiotic stresses but also by *E. necator*. The small size and distinct expression profiles of the grapevine *DHN* gene family makes it an excellent model to elucidate functional differentiation within this gene family, which should contribute to the further understanding of these genes in plants.

## Methods

### Plant materials

*V. yeshanensis* acc. Yanshan-1 and *V. vinifera* cv. Pinot Noir were obtained from the Grapevine Repository of Northwest A&F University, Yangling, Shaanxi, China. One-year old rooted seedlings of Yanshan-1 and Pinot Noir were maintained in a greenhouse, and were utilized for stress experiments. For expression analysis in different plant tissues, root, stem, leaf, seed and fruit peel samples were harvested from three representative veraison-stage *V. vinifera* and *V. yeshanensis* plants, respectively, that had been grown in the field. For expression analysis during seed development, flower buds were harvested from three Pinot Noir plants grown in the field 6 days before flower opening (−6 daf). Flowers were collected at flower opening (0 daf) and young berries were harvested at 6 daf. In addition, seeds were collected from 12–66 daf. Samples were frozen in liquid nitrogen and stored at −80°C.

### RNA extraction and first-strand cDNA synthesis

Total RNA was isolated from plant tissues using the method described by Reid et al. [61]. Subsequently, RNA was treated with 10 units RQ1 RNase-free DNase (Promega, Shanghai, China) in the presence of 100 units RNase inhibitor at 37°C for 30 min, followed by extraction with phenol:chloroform:isoamyl alcohol (25:24:1) and chloroform:isoamyl alcohol (24:1). RNA was then precipitated with ethanol and dissolved in RNase-free water. First-strand cDNA synthesis was carried out using 1 μg total RNA and the PrimeScript™ RT reagent Kit (TaKaRa Biotechnology, Dalian, China) with 2.5 μM oligo dT primer and 2.5 μM random 6mer primer. The reaction mixture was incubated at 37°C for 40 min and the reverse transcriptase was then inactivated at 85°C for 5 s.

### Cloning of *DHN* genes from *V. yeshanensis* and *V. vinifera*

A fragment of the *VyDHN1* coding sequence was amplified from drought-treated leaves of *V. yeshanensis* acc. Yanshan-1 using differential display reverse-transcription PCR (DDRT-PCR) as described by Lin et al. [62]. Briefly, first-strand cDNA was synthesized from 1 μg total RNA isolated from leaves subjected to drought at 0, 2, 3, 4, and 5 d following onset of treatment using primer T_11_VA (V = A, C, G) at 37°C for 1 h with 200 units of M-MLV (Promega) according to the manufacturer’s instructions. This was followed by PCR amplification with primers T_11_VA and S476 (CCAAGCTGCC), followed by separation on a 6% polyacrylamide gel. Differential fragments were recovered, amplified by a second round of PCR using the same parameters as the first, and cloned into the pGEM-T easy vector (Promega). The 5’ end of the *VyDHN1* cDNA was obtained by 5’ rapid amplification of cDNA ends (5’ RACE) using the BD SMART RACE cDNA Amplification Kit (Clontech, CA, USA) with primer VD1-GSP1 (see Additional file 3 for primer sequence) according to the manufacturer’s recommendations. The full-length *VyDHN1* sequence was then deduced through alignment of the original DDRT-PCR fragment and 5’ RACE sequence.

The encoded protein sequence of the *VyDHN1* gene was used to identify four *V. vinifera DHN* genes containing putative K-segments via BLAST analyses. BLAST analyses were also performed against the predicted grapevine *DHN* genes using ten previously identified Arabidopsis DHN proteins as query sequences [18]. These results were further validated by searching for *Vitis* DHN sequences in the Pfam database [63].

For the remaining *DHN* genes, seeds were harvested from *V. yeshanensis* at veraison. The *DHN* genes were amplified from cDNA using PrimeSTAR® HS DNA polymerase (TaKaRa) with primers that were designed based upon the *DHN* genes of *V. vinifera* cv. Pinot Noir (see Additional file 3). Cycling parameters for amplification of *VyDHN2* and *VyDHN4* were as follows: 94°C for 3 min, 25 cycles at 94°C for 30 s, 60°C for 30 s and 72°C for 30 s, followed by a final elongation at 72°C for 5 min. The same parameters were utilized in the case of *VyDHN3* except that 40 cycles were necessary for its amplification. Subsequently, all four *V. yeshanensis DHN* genes were also amplified from genomic DNA to identify intronic regions using the same primers.

*V. yeshanensis DHN* promoters, including 1500-bp of upstream sequence in each case, were amplified from genomic DNA using PrimeSTAR® HS DNA polymerase (TaKaRa) with primers that were designed based upon the *DHN* genes of *V. vinifera* cv. Pinot Noir (see Additional file 4). Cycling parameters were as follows: 94°C for 3 min, 30 cycles at 94°C for 30 s, 60°C for 30 s and 72°C for 2-3 min, followed by a final elongation at 72°C for 5 min. PCR products were cloned into pGEM-T easy (Promoga) and three clones were sequenced per *DHN* gene.

### Treatment of plants with various hormones, as well as abiotic and biotic stress

Drought experiments were conducted by withholding water from *V. yeshanensis* and *V. vinifera* seedlings. Leaves were harvested 0, 0.5, 1, 2, 3, 4, 5, and 6 d following onset of treatment. Subsequently, the stressed plants were watered to soil saturation and leaves were collected 0.25, 0.5, 2, 6, 12, 24, and 48 h after watering. Plants grown under a normal watering regime were used as a control. For cold- and heat-stress induction, *V. yeshanensis* and *V. vinifera* seedlings were maintained in a growth chamber at either 4°C or 40°C and leaves were harvested 0, 2, 6, 12, 24 and 48 h after treatment. Mock-treated control plants were kept in a growth chamber at 22°C. Pathogen treatment was carried out by inoculating the leaves of *V. yeshanensis* and *V. vinifera* with *E. necator* as previously described [27]. Prior to inoculation, leaves were sprayed with sterile water, and leaves were harvested 0, 0.5, 1, 2, 3, 4, 5, and 6 d after inoculation. Control plants simply underwent the sterile water spray and were not inoculated.

For signaling molecule treatment, 100 μM ABA [29], 100 μM SA [64], and 50 μM MeJA [65] were sprayed on the leaves of *V. vinifera* and leaf samples were harvested 0, 1, 2, 4, 6, 8 h post-treatment. Leaves sprayed with 0.05% Tween 20 solution were used as a negative control. All stress-induction experiments were performed on three independent plants for each treatment.

### Semi-quantitative and real-time RT-PCR analysis

Semi-quantitative RT-PCR was performed using Premix Ex Taq® Version2.0 (TaKaRa) and *DHN*-specific primers that were designed to anneal to either side of an intron in both grapevine species (see Additional file 5 for primer sequences). Genomic DNA was utilized as a size control for unspliced transcripts. Reactions lacking reverse transcriptase were utilized as a negative control to exclude DNA contamination. Each experimental reaction (25 μl final volume) contained 1 μl of template cDNA along with 400 nM of each primer. Cycling parameters were as follows: 94°C for 3 min, 25 cycles at 94°C for 30 s, 60°C for 30 s and 72°C for 30 s, followed by a final elongation at 72°C for 5 min. The *actin1* transcript [GenBank:AF369524] was utilized as an internal control using primers designed to anneal to both *V. vinifera* and *V. yeshanensis* sequences and the same general parameters as the *DHN* transcripts, except 20 cycles were utilized for amplification. PCR products were separated on a 1.5% agarose gel containing ethidium bromide, and photographed using a Bio Imaging System (Syngene, Cambridge, UK). At least two technical replications were conducted in each case.

Real-time quantitative RT-PCR was conducted via the ΔΔC_T_ method using the SYBR® Premix *Ex Taq* II Kit (TaKaRa) with primer pairs designed to anneal within the second exon of each *DHN* gene in both grapevine species (see Additional file 6 for primer sequences). The reactions were carried out in triplicate using 1 μl template cDNA in a final volume of 25 μl in an iQ5 Real Time PCR System (Bio-Rad, CA, USA) with the following thermal parameters: 95°C for 10 s, followed by 40 cycles of 94°C for 5 s and 60°C for 30 s, with a final melting gradient from 60°C to 95°C at a rate of 1°C per min. The grapevine *actin1* gene was utilized as an internal control. Relative expression levels of each *DHN* gene from both species were analyzed using the IQ5 software and were denoted as the fold-difference from expression present at baseline levels. Paired t-tests were conducted using Origin Pro 8.0 software (OriginLab Corp., Northampton, MA, USA) to assess the significance of expression level differences between treated samples and the mock controls. Differences were deemed significant at p < 0.05. Baseline expression signifies that which was present prior to treatment (ie. the first time point where relative expression is set to 1), except in the case of *DHN3*, where no expression was noted until 18 daf and therefore this time point was utilized as the baseline.

### *In silico* analysis of *DHN* genes and their encoded proteins

Chromosomal locations of *VvDHN* genes were predicted using the BLAT server through the Genoscope Genome Browser (http://www.genoscope.cns.fr/blat-server/cgi-bin/vitis/webBlat). To identify putative *cis*-acting elements within the *V. vinifera DHN* promoters, contigs containing the respective genes were obtained using BLAST. Intergenic regions between the *DHN* genes and their upstream genes were determined according to annotations provided in GenBank. In the case of *V. yeshanensis DHN* promoters, sequences were obtained directly by cloning. The presence of regulatory elements in 1500-bp of sequence upstream of each translational start codon was determined using the PlantCARE database [36].

Protein MW (molecular weight), pI (isoelectric point) and GRAVY (grand average of hydropathy) were predicted using the ProtParam program (Expasy tools) based on their amino acid compositions. Predictions of intrinsic disorder within each *DHN* gene from both species were conducted using the PONDR-FIT tool [66]. Protein secondary structures were predicted using the PSIPRED v3.0 program [67]. The sequence algorithm NetPhosK (Expasy), with its probability limit set to 60%, was utilized to predict phosphorylation sites in VvDHN and VyDHN proteins.

Phylogenetic analysis was carried out by performing multiple alignments of full-length DHN protein sequences from *V. viniferaV. yeshanensisH. vulgare* and Arabidopsis using MEGA5 [68]. DHN sequences from Arabidopsis and barley were obtained from previous reports [18,32-34]. An unrooted dendrogram was constructed based on the alignment with PhyML using the maximum likelihood method [69].

## Abbreviations

ABA, Abscisic acid; ABRE, ABA-responsive element; CK2, Casein kinase 2; Daf, Days after flowering; DDRT-PCR, Differential display reverse-transcription PCR; DHN, Dehydrin; Dpi, Days post inoculation; DRE, Dehydration-responsive element; HSE, Heat shock-responsive element; LTR, Low temperature-responsive element; MeJA, Methyl jasmonate; MeJA-RE, Methyl jasmonate-responsive element; NLS, Nuclear localization signal; PKC, Protein kinase C; qRT-PCR, Quantitative reverse-transcription PCR; SA, Salicylic acid; SnRK, Snf1-related kinase.

## Authors’ contributions

YY contributed to the design of the study, conducted the majority of experiments and drafted the manuscript; MH contributed to the powdery mildew treatment experiment; ZZ contributed to the signaling molecule treatment experiment; SL contributed to the seed development experiment; YX and CZ were involved in the design of the study and preparation of the manuscript; SDS participated in analysis of results and preparation of the manuscript; YW conceived, designed and directed the study and contributed to the preparation of the manuscript. All authors read and approved the final manuscript.

## Supplementary Material

Additional file 1 **Cloning of the* DHN1 *coding sequence from drought-treated leaves of* V. yeshanensis *.** Click here for file

Additional file 2 **Structure prediction of DHN proteins from* V. yeshanensis *and* V. vinifera *.** Click here for file

Additional file 3 **Sequence of primers used for cloning* DHN *genes in grapevine**. Click here for file

Additional file 4 **Sequence of primers used for cloning* DHN *promoters from* V. yeshanensis ***. Click here for file

Additional file 5 Sequence of primers used for semi-quantitative RT-PCR in grapevine. Click here for file

Additional file 6 Sequence of primers used for real-time qRT-PCR in grapevine. Click here for file

## References

[B1] Nylander M Svensson J Palva ET Welin BV Stress-induced accumulation and tissue-specific localization of dehydrins in Arabidopsis thaliana Plant Mol Biol 2001 45 263 279 10.1023/A:1006469128280 11292073

[B2] Xu J Zhang Y Guan Z Wei W Han L Chai T Expression and function of two dehydrins under environmental stresses in Brassica juncea L Mol Breeding 2008 21 431 438 10.1007/s11032-007-9143-5

[B3] Kim SY Nam KH Physiological roles of ERD10 in abiotic stresses and seed germination of Arabidopsis Plant Cell Rep 2010 29 203 209 10.1007/s00299-009-0813-0 20054552

[B4] Puhakainen T Hess MW Kela PM Svensson J Heino P Palva ET Overexpression of multiple dehydrin genes enhances tolerance to freezing Plant Mol Biol 2004 54 743 753 15356392 10.1023/B:PLAN.0000040903.66496.a4

[B5] Peng Y Reyes JL Wei H Yang Y Karlson D Covarrubias AA Krebs SL Fessehaie A Arora R RcDhn5, a cold acclimation-responsive dehydrin from Rhododendron catawbiense rescues enzyme activity from dehydration effects in vitro and enhances freezing tolerance in RcDhn5-overexpressing Arabidopsis plants Physiol Plant 2008 134 583 597 10.1111/j.1399-3054.2008.01164.x 19000195

[B6] Shekhawat UKS Srinivas L Ganapathi TR MusaDHN-1, a novel multiple stress-inducible SK3-type dehydrin gene, contributes affirmatively to drought- and salt-stress tolerance in banana Planta 2011 10.1007/s00425-011-1455-3 21671068

[B7] Ochoa-Alfaro AE Rodríguez-Kessler M Pérez-Morales MB Delgado-Sánchez P Cuevas-Velazquez CL Gómez-Anduro G Jiménez-Bremont JF Functional characterization of an acidic SK3 dehydrin isolated from an Opuntia streptacantha cDNA library Planta 2011 10.1007/s00425-011-1531-8 21984262

[B8] Hundertmark M Buitink J Leprince O Hincha DK The reduction of seed-specific dehydrins reduces seed longevity in Arabidopsis thaliana Seed Sci Res 2011 21 165 173 10.1017/S0960258511000079

[B9] Eriksson SK Harryson P D Luttge U, Beck E, Bartels D Molecular biology, structure and function Plant Desiccation Tolerance 2011 Springer-Verlag, Berlin 289 305

[B10] Kovacs D Kalmar E Torok Z Tompa P Chaperone activity of ERD10 and ERD14, two disordered stress-related plant proteins Plant Physiol 2008 147 381 390 10.1104/pp.108.118208 18359842 PMC2330285

[B11] Brini F Yamamoto A Jlaiel L Takeda S Hobo T Dinh HQ Hattori T Masmoudi K Hanin M Pleiotropic effects of the wheat dehydrin DHN-5 on stress responses in Arabidopsis Plant Cell Physiol 2011 52 676 688 10.1093/pcp/pcr030 21421569

[B12] Close TJ Dehydrins: Emergence of a biochemical role of a family of plant dehydration proteins Physiol Plant 1996 97 795 803 10.1111/j.1399-3054.1996.tb00546.x

[B13] Koag MC Wilkens S Fenton RD Resnik J Vo E Close TJ The K-segment of maize DHN1 mediates binding to anionic phospholipid vesicles and concomitant structural changes Plant Physiol 2009 150 1503 1514 10.1104/pp.109.136697 19439573 PMC2705017

[B14] Hughes S Graether SP Cryoprotective mechanism of a small intrinsically disordered dehydrin protein Protein Sci 2011 20 42 50 10.1002/pro.534 21031484 PMC3047060

[B15] Jensen A Goday A Figueras M Jessop A Pages M Phosphorylation mediates the nuclear targeting of the maize Rab17 protein Plant J 1998 13 691 697 10.1046/j.1365-313X.1998.00069.x 9681011

[B16] Riera M Figueras M Lopez C Goday A Pages M Protein kinase CK2 modulates developmental functions of the abscisic acid responsive protein Rab17 from maize Proc Natl Acad Sci USA 2004 101 9879 9884 10.1073/pnas.0306154101 15159549 PMC470767

[B17] Alsheikh MK SVensson J Randall SK Phosphorylation regulated ion-binding is a property shared by the acidic subclass dehydrins Plant Cell Environ 2005 28 1114 1122 10.1111/j.1365-3040.2005.01348.x

[B18] Hundertmark M Hincha DK LEA (Late Embryogenesis Abundant) proteins and their encoding genes in Arabidopsis thaliana BMC Genomics 2008 9 118 10.1186/1471-2164-9-118 18318901 PMC2292704

[B19] Hara M Shinoda Y Tanaka Y Kuboi T DNA binding of citrus dehydrin promoted by zinc ion Plant Cell Environ 2009 32 532 541 10.1111/j.1365-3040.2009.01947.x 19183287

[B20] Goday A Jensen AB Culianez-Macia FA Alba MM Figueras M Serratosa J Torrent M Pages M The maize abscisic acid-responsive protein Rab17 is located in the nucleus and lnteracts with nuclear localization signals Plant Cell 1994 6 351 360 8180497 10.1105/tpc.6.3.351 PMC160438

[B21] Rahman LN Smith GST Bamm VV Voyer-Grant JAM Moffatt BA Dutcher JR Harauz G Phosphorylation of Thellungiella salsuginea dehydrins TsDHN-1 and TsDHN-2 facilitates cation-Induced conformational changes and actin assembly Biochemistry 2011 50 9587 9604 10.1021/bi201205m 21970344

[B22] Eriksson SK Kutzer M Procek J Gröbner G Harryson P Tunable membrane binding of the intrinsically disordered dehydrin Lti30, a cold-induced plant stress protein Plant Cell 2011 23 2391 2404 10.1105/tpc.111.085183 21665998 PMC3160030

[B23] Tommasini L Svensson JT Rodriguez EM Wahid A Malatrasi M Kato K Wanamaker S Resnik J Close TJ Dehydrin gene expression provides an indicator of low temperature and drought stress: transcriptome-based analysis of Barley (Hordeum vulgare L.) Funct Integr Genomics 2008 8 387 405 10.1007/s10142-008-0081-z 18512091

[B24] Tripepi M Pöhlschroder M Bitonti MB Diversity of dehydrins in Oleae europaea plants eposed to sress Open Plant Sci J 2011 5 9 13 10.2174/1874294701105010009

[B25] He P Niu L Study of cold hardiness in the wild Vitis native to China Acta Horticulturae Sinica 1989 16 81 88 in Chinese with English abstract

[B26] Wang Y Yang Y Zhang J Pan X Wan Y Preliminary identification of drought resistance of Chinese wild Vitis species and its interspecific hybrids Acta Horticulturae Sinica 2004 31 711 714 in Chinese with English abstract

[B27] Wang Y Liu Y He P Chen J Lamicanra O Lu J Evaluation of foliar resistance to Uncinula necator in Chinese wild Vitis species Vitis 1995 34 159 164

[B28] Cramer GR Ergul A Grimplet J Tillett RL Tattersall EAR Bohlman MC Vincent D Sonderegger J Evans J Osborne C Water and salinity stress in grapevines: early and late changes in transcript and metabolite profiles Funct Integr Genomics 2007 7 111 134 10.1007/s10142-006-0039-y 17136344

[B29] Xiao H Nassuth A Stress- and development-induced expression of spliced and unspliced transcripts from two highly similar dehydrin 1 genes in V. riparia and V. vinifera Plant Cell Rep 2006 25 968 977 10.1007/s00299-006-0151-4 16552595

[B30] Jaillon O Aury J-M Noel B Policriti A Clepet C Casagrande A Choisne N Aubourg S Vitulo N Jubin C The grapevine genome sequence suggests ancestral hexaploidization in major angiosperm phyla Nature 2007 449 463 467 10.1038/nature06148 17721507

[B31] Vlad F Turk BE Peynot P Leung J Merlot S A versatile strategy to define the phosphorylation preferences of plant protein kinases and screen for putative substrates Plant J 2008 55 104 117 10.1111/j.1365-313X.2008.03488.x 18363786

[B32] Choi D-W Zhu B Close TJ The barley (Hordeum vulgare L.) dehydrin multigene family: sequences, allele types, chromosome assignments, and expression characteristics of 11 Dhn genes of cv Dicktoo Theor Appl Genet 1999 98 1234 1247 10.1007/s001220051189

[B33] Choi DW Close TJ A newly identified barley gene, Dhn12, encoding a YSK2 DHN, is located on chromosome 6 H and has embryo-specific expression Theor Appl Genet 2000 100 1274 1278 10.1007/s001220051434

[B34] Rodriguez EM Svensson JT Malatrasi M Choi DW Close TJ Barley Dhn13 encodes a KS-type dehydrin with constitutive and stress responsive expression Theor Appl Genet 2005 110 852 858 10.1007/s00122-004-1877-4 15711789

[B35] Bies-Etheve N Gaubier-Comella P Debures A Lasserre E Jobet E Raynal M Cooke R Delseny M Inventory, evolution and expression profiling diversity of the LEA (late embryogenesis abundant) protein gene family in Arabidopsis thaliana Plant Mol Biol 2008 67 107 124 10.1007/s11103-008-9304-x 18265943

[B36] Lescot M Dehais P Thijs G Marchal K Moreau Y Peer YV Rouze P Rombauts S PlantCARE, a database of plant cis-acting regulatory elements and a portal to tools for in silico analysis of promoter sequences Nucleic Acids Res 2002 30 325 327 10.1093/nar/30.1.325 11752327 PMC99092

[B37] Baker SS Wilhelm KS Thomashow MF The 5'-region of Arabidopsis thaliana cor15a has cis-acting elements that confer cold-, drought- and ABA-regulated gene expression Plant Mol Biol 1994 24 701 713 10.1007/BF00029852 8193295

[B38] Busk PK Jensen AB Pages M Regulatory elements in vivo in the promoter of the abscisic acid responsive gene rab17 from maize Plant J 1997 11 1285 1295 10.1046/j.1365-313X.1997.11061285.x 9225468

[B39] Pastuglia M Roby D Dumas C Cock JM Rapid induction by wounding and bacterial infection of an S gene family receptor-like kinase in Brassica oleracea Plant Cell 1997 9 1 13 10.1105/tpc.9.1.49 PMC156900 9014364

[B40] White AJ Dunn MA Brown K Hughes MA Comparative analysis of genomic sequence and expression of a lipid transfer protein gene family in winter barley J Exp Bot 1994 45 1885 1892 10.1093/jxb/45.12.1885

[B41] Diaz-De-Leon F Klotz KL Lagrimini M Nucleotide sequence of the tobacco (Nicotiana tabacum) anionic peroxidase gene Plant Physiol 1993 101 1117 1118 10.1104/pp.101.3.1117 8310051 PMC158735

[B42] Rouster J Leah R Mundy J Cameron-Mills V Identification of a methyl jasmonate-responsive region in the promoter of a lipoxygenase 1 gene expressed in barley grain Plant J 1997 11 513 523 10.1046/j.1365-313X.1997.11030513.x 9107039

[B43] Hennig J Dewey RE Cutt JR Klessig DF Pathogen, salicylic acid and developmental dependent expression of a beta-1,3-glucanase/GUS gene fusion in transgenic tobacco plants Plant J 1993 4 481 493 10.1046/j.1365-313X.1993.04030481.x 8220491

[B44] Takaiwa F Oono K Wing D Kato A Sequence of three members and expression of a new major subfamily of glutelin genes from rice Plant Mol Biol 1991 17 875 885 10.1007/BF00037068 1680490

[B45] Wang X Zhu H Jin G Liu H Wu W Zhu J Genome-scale identification and analysis of LEA genes in rice (Oryza sativa L.) Plant Sci 2007 172 414 420 10.1016/j.plantsci.2006.10.004

[B46] Liu C-C Li C-M Liu B-G Ge S-J Dong X-M Li W Zhu H-Y Wang B-C Yang C-P Genome-wide identification and characterization of a dehydrin gene family in poplar (Populus trichocarpa) Plant Mol Biol Rep 2012

[B47] Velasco R Zharkikh A Troggio M Cartwright DA Cestaro A Pruss D Pindo M FitzGerald LM Vezzulli S Reid J A high quality draft consensus sequence of the genome of a heterozygous grapevine variety PLoS One 2007 2 e1326 10.1371/journal.pone.0001326 18094749 PMC2147077

[B48] Lopez CG Banowetz GM Peterson CJ Kronstad WE Dehydrin expression and drought tolerance in seven wheat cultivars Crop Sci 2003 43 577 582 10.2135/cropsci2003.0577

[B49] Suprunova T Krugman T Fahima T Chen G Shams I Korol A Nevo E Differential expression of dehydrin genes in wild barley, Hordeum spontaneum, associated with resistance to water deficit Plant Cell Environ 2004 27 1297 1308 10.1111/j.1365-3040.2004.01237.x

[B50] Qian G Liu Y Ao D Yang F Yu M Differential expression of dehydrin genes in hull-less barley (Hordeum vulgare ssp. vulgare) depending on duration of dehydration stress Can J Plant Sci 2008 88 899 906 10.4141/CJPS08015

[B51] Hu L Wang Z Du H Huang B Differential accumulation of dehydrins in response to water stress for hybrid and common bermudagrass genotypes differing in drought tolerance J Plant Physiol 2010 167 103 109 10.1016/j.jplph.2009.07.008 19716198

[B52] Bernacchia G Salamini F Bartels D Molecular characterization of the rehydration process in the resurrection plant Craterostigma plantagineum Plant Physiol 1996 111 1043 12226346 10.1104/pp.111.4.1043 PMC160978

[B53] Wisniewski ME Bassett CL Renaut J Robert Farrell J Tworkoski T Artlip TS Differential regulation of two dehydrin genes from peach (Prunus persica) by photoperiod, low temperature and water deficit Tree Physiol 2006 26 575 584 10.1093/treephys/26.5.575 16452071

[B54] Layton BE Boyd MB Tripepi MS Bitonti BM Dollahon MNR Balsamo RA Dehydration-induced expression of a 31-kDa dehydrin in Polypodium polypodioides (Polypodiaceae) may enable large, reversible deformation of cell walls Am J Bot 2010 97 535 544 10.3732/ajb.0900285 21622416

[B55] Dobra J Vankova R Havlova M Burman AJ Libus J Storchova H Tobacco leaves and roots differ in the expression of proline metabolism-related genes in the course of drought stress and subsequent recovery J Plant Physiol 2011 168 1588 1597 10.1016/j.jplph.2011.02.009 21481968

[B56] Lovisolo C Perrone I Hartung W Schubert A An abscisic acid-related reduced transpiration promotes gradual embolism repair when grapevines are rehydrated after drought New Phytol 2008 180 642 651 10.1111/j.1469-8137.2008.02592.x 18700860

[B57] Liu B Zhang S Zhu X Yang Q Wu S Mei M Mauleon R Leach J Mew T Leung H Candidate defense genes as predictors of quantitative blast resistance in rice Mol Plant Microbe Interact 2004 17 1146 1152 10.1094/MPMI.2004.17.10.1146 15497407

[B58] Pieterse CMJ Leon-Reyes A Van der Ent S Van Wees SCM Networking by small-molecule hormones in plant immunity Nature Chem Biol 2009 5 308 316 10.1038/nchembio.164 19377457

[B59] Yamaguchishinozaki K Shinozaki K Organization of cis-acting regulatory elements in osmotic- and cold-stress-responsive promoters Trends Plant Sci 2005 10 88 94 10.1016/j.tplants.2004.12.012 15708346

[B60] Washida H Wu C-Y Suzuki A Yamanouchi U Akihama T Harada K Takaiwa F Identification of cis-regulatory elements required for endosperm expression of the rice storage protein glutelin gene GluB-1 Plant Mol Biol 1999 40 1 12 10.1023/A:1026459229671 10394940

[B61] Reid KE Olsson N Schlosser J Peng F Lund ST An optimized grapevine RNA isolation procedure and statistical determination of reference genes for real-time RT-PCR during berry development BMC Plant Biol 2006 6 27 10.1186/1471-2229-6-27 17105665 PMC1654153

[B62] Lin L Wang X Wang Y cDNA clone, fusion expression and purification of the novel gene related to ascorbate peroxidase from Chinese wild Vitis pseudoreticulata in E. coli Mol Biol Rep 2006 33 197 206 10.1007/s11033-006-0008-5 16850189

[B63] Finn RD Mistry J Tate J Coggill P Heger A Pollington JE Gavin OL Gunasekaran P Ceric G Forslund K The Pfam protein families database Nucleic Acids Res 2010 38 Database issue D211 D222 19920124 10.1093/nar/gkp985 PMC2808889

[B64] Wang L-J Li S-H Thermotolerance and related antioxidant enzyme activities induced by heat acclimation and salicylic acid in grape (Vitis vinifera L.) leaves Plant growth reg 2006 48 137 144 10.1007/s10725-005-6146-2

[B65] Repka V Fischerova I Silharova K Methyl jasmonate is a potent elicitor of multiple defense responses in grapevine leaves and cell-suspension cultures Biol Plant 2004 48 273 283

[B66] Xue B Dunbrack RL Williams RW Dunker AK Uversky VN PONDR-FIT: A meta-predictor of intrinsically disordered amino acids Biochim Biophys Acta 2010 1804 996 1010 10.1016/j.bbapap.2010.01.011 20100603 PMC2882806

[B67] Bryson K McGuffin LJ Marsden RL Ward JJ Sodhi JS Jones DT Protein structure prediction servers at University College London Nucl Acids Res 2005 33 Web Server issue W36 38 15980489 10.1093/nar/gki410 PMC1160171

[B68] Tamura K Peterson D Peterson N Stecher G Nei M Kumar S MEGA5: Molecular evolutionary genetics analysis using maximum likelihood, evolutionary distance, and maximum parsimony methods Mol Biol Evol 2011 28 2731 2739 10.1093/molbev/msr121 21546353 PMC3203626

[B69] Guindon S Gascuel O A simple, fast and accurate algorithm to estimate large phylogenies by maximum likelihood Syst Biol 2003 52 696 704 10.1080/10635150390235520 14530136

