# Supplementary material for: Identification of the dehydrin gene family from grapevine species and analysis of their responsiveness to various forms of abiotic and biotic stress
Source: BMC Plant Biol. 2012 Aug 10;12:140. doi: 10.1186/1471-2229-12-140 (PMC3460772; doi:10.1186/1471-2229-12-140)
Supplement: Additional file 1 — Cloning of the DHN1 coding sequence from drought-treated leaves of V. yeshanensis . [file 1471-2229-12-140-S1.doc]

**
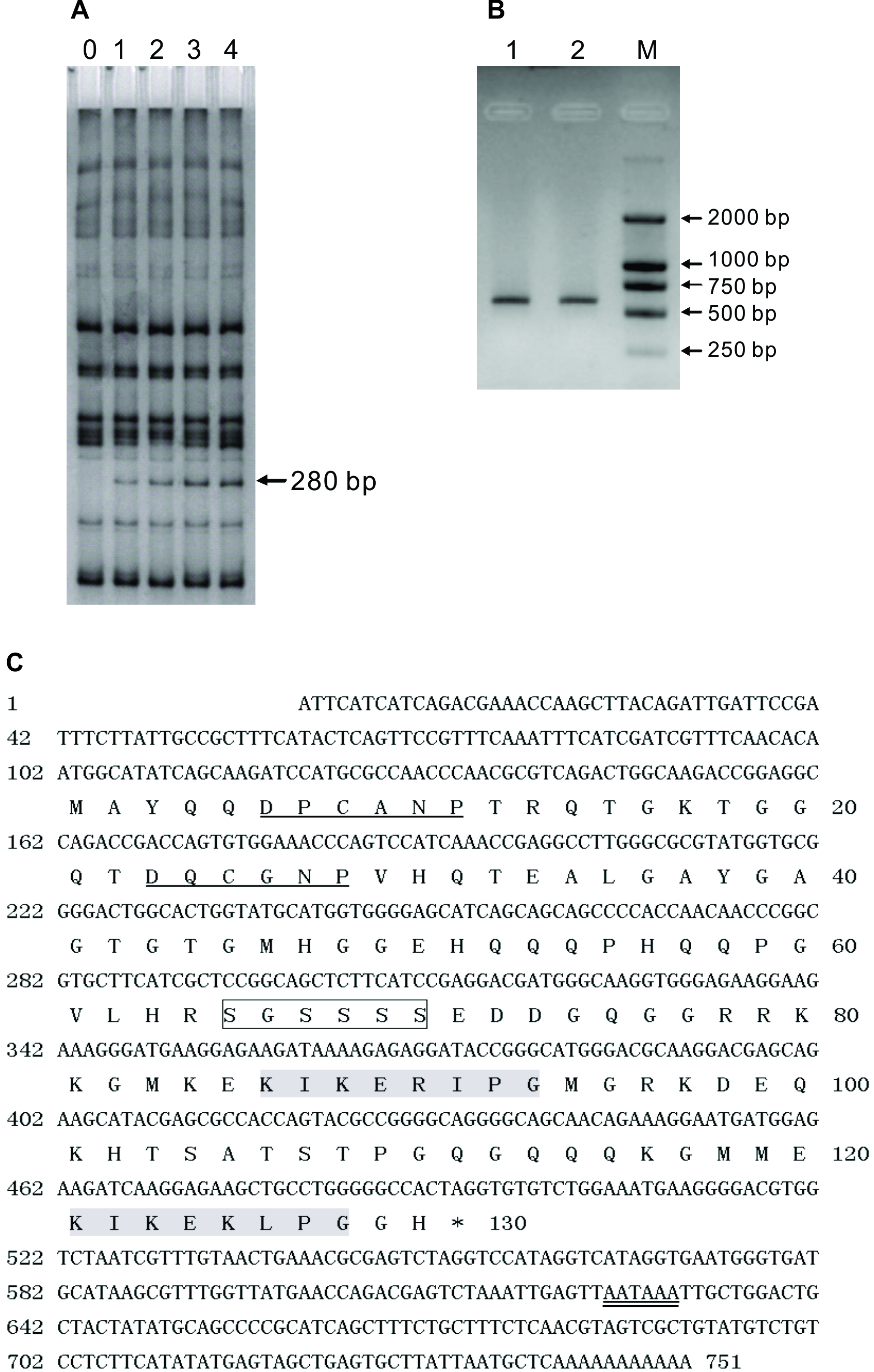
**

**Additional file 1 Cloning of the *DHN1* coding sequence from drought-treated leaves of *V. yeshanensis*.**

A. A 280-bp fragment of the *VyDHN1* cDNA was obtained by DDRT-PCR. RNA samples were extracted from *V. yeshanensis* leaves treated with drought for 0, 2, 3, 4 and 5 d. DDRT-PCR fragments were separated on a 6% polyacrylamide gel and the differential band from drought-treated samples was isolated and cloned. B. 5’RACE of the *VyDHN1* gene fragment. 5’RACE was carried out using the BD SMART^TM^ RACE kit (Clontech) and primer VD1-GSP1. C. The full-length *VyDHN1* gene was obtained through alignment of the DDRT-PCR and 5’RACE fragments [GenBank:JF900497]. Two regions exhibiting homology to Y-segments are underlined, the putative S-segment is boxed, and the two putative K-segments are shaded. The putative polyadenylation signal sequence is underlined with a double line.
