# Supplementary material for: Identification of the dehydrin gene family from grapevine species and analysis of their responsiveness to various forms of abiotic and biotic stress
Source: BMC Plant Biol. 2012 Aug 10;12:140. doi: 10.1186/1471-2229-12-140 (PMC3460772; doi:10.1186/1471-2229-12-140)
Supplement: Additional file 2 — Structure prediction of DHN proteins from V. yeshanensis and V. vinifera . [file 1471-2229-12-140-S2.doc]

**
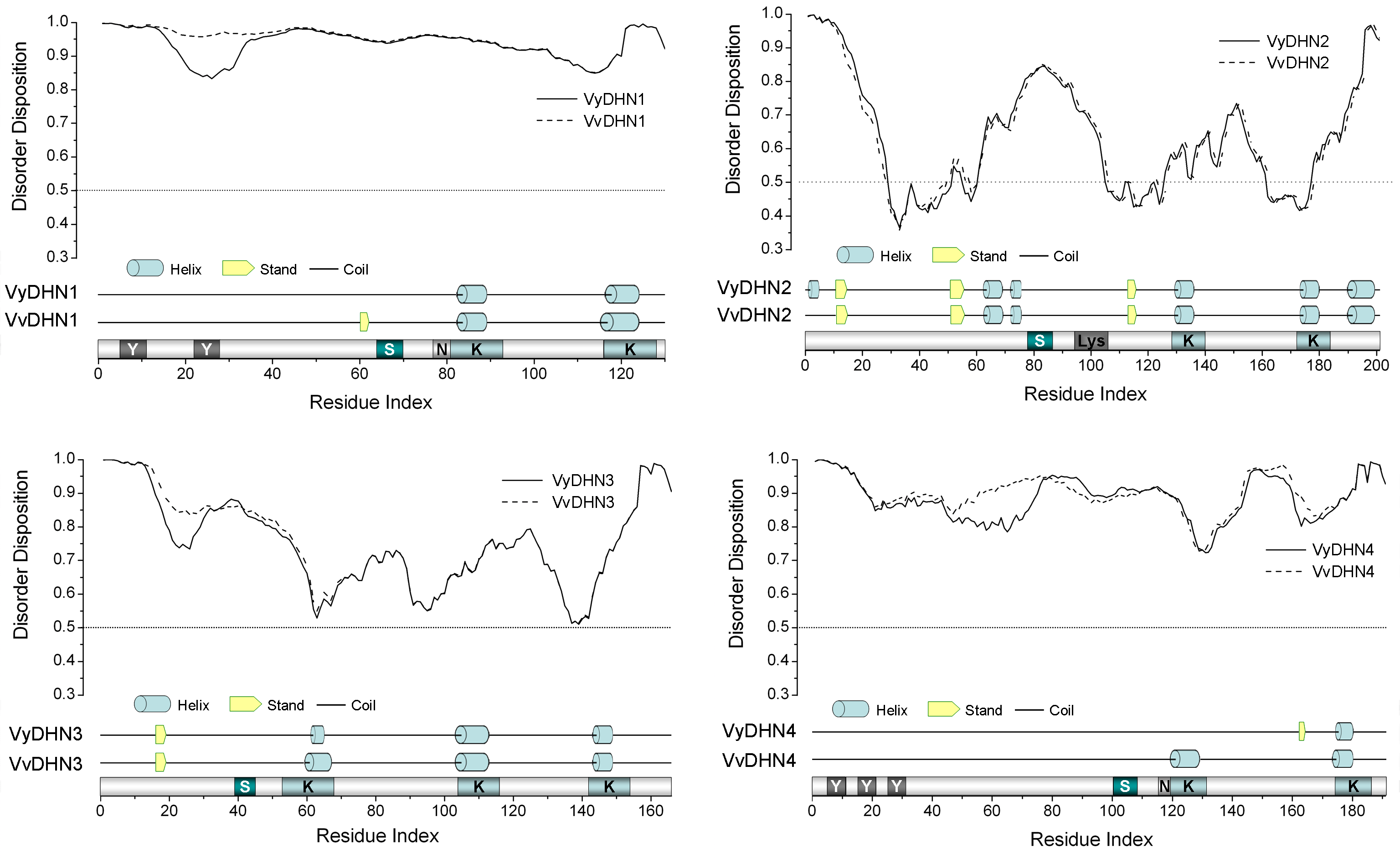
**

**Additional file 2 Structure prediction of DHN proteins from *V. yeshanensis* and *V. vinifera.***

The graphs show predicted protein disorder for each grapevine DHN protein. Beneath each graph is a diagram displaying the predicted secondary structure of each protein. Also included are schematic diagrams showing the different domains of each encoded protein sequence. Y: Y-segment; S: S-segment; K: K-segment; N: nuclear localization signal; Lys: Lysine-rich segment.
