# Supplementary material for: Identification of the dehydrin gene family from grapevine species and analysis of their responsiveness to various forms of abiotic and biotic stress
Source: BMC Plant Biol. 2012 Aug 10;12:140. doi: 10.1186/1471-2229-12-140 (PMC3460772; doi:10.1186/1471-2229-12-140)
Supplement: Additional file 3 — Sequence of primers used for cloning DHN genes in grapevine. [file 1471-2229-12-140-S3.doc]

**Additional file 3 Sequence of primers used for cloning *DHN*** genes in grapevine.

| Gene | Primer pair | Forward primer sequence | Reverse primer sequence | Product (bp) |
| --- | --- | --- | --- | --- |
| DHN1 | VD1-GSP1^*^ | TCCCCTTCATTTCCAGACACACCT |  |  |
| DHN2 | VD2mF/VD2mR | TGATTGCATTTTTCTGAAGGCT | AACAAACCGCACAATCAACGA | 727 |
| DHN3 | VD3mF/VD3mR | CTAACAAAATGGCAGATCAGCA | AGTCCCCTAATCAGTTTCCATC | 531 |
| DHN4 | VD4mF/VD4mR | TTGTGGGTCTATAGATTTTGCT | TAGCAACGAAGCCATTTGTACA | 636 |

^*^ This primer was used for 5’ RACE of the *DHN1* gene.
