# Supplementary material for: Identification of the dehydrin gene family from grapevine species and analysis of their responsiveness to various forms of abiotic and biotic stress
Source: BMC Plant Biol. 2012 Aug 10;12:140. doi: 10.1186/1471-2229-12-140 (PMC3460772; doi:10.1186/1471-2229-12-140)
Supplement: Additional file 4 — Sequence of primers used for cloning DHN promoters from V. yeshanensis . [file 1471-2229-12-140-S4.doc]

**Additional file 4 Sequence of primers used for cloning *DHN* promoters from *V. yeshanensis***.

| Gene | Primer pair | Forward primer sequence | Reverse primer sequence |
| --- | --- | --- | --- |
| *DHN1* | VD1PF/VD1PR | CTGGGCTCACTGGACTCTTAGACG | ACTGAGTATGAAAGCGGCAATAAG |
| *DHN2* | VD2PF/VD2PR | GCACTACCTTTTTGATGTGAGTG | CCACGATCCTTGGTCTCGACG |
| *DHN3* | VD3PF/VD3PR | CTCAGTGTCCTGTTAGTTGCTGC | CGAACTAGAGCTGCTATGATGCTG |
| *DHN4* | VD4PF/VD4PR | AACTGTATTCGCCTCTTAAATCCT | CGTGCTCGTCGGTTAACTGAACTG |
