# Supplementary material for: Identification of the dehydrin gene family from grapevine species and analysis of their responsiveness to various forms of abiotic and biotic stress
Source: BMC Plant Biol. 2012 Aug 10;12:140. doi: 10.1186/1471-2229-12-140 (PMC3460772; doi:10.1186/1471-2229-12-140)
Supplement: Additional file 5 — Sequence of primers used for semi-quantitative RT-PCR in grapevine. [file 1471-2229-12-140-S5.doc]

**Additional file 5** Sequence of primers used for semi-quantitative RT-PCR in grapevine.

| Gene | Primer pair | Forward primer sequence | Reverse primer sequence | Product^*^ (bp) |
| --- | --- | --- | --- | --- |
| DHN1 | VD1SF/VD1SR | AACCCAGTCCATCAAACCGA | GACCTATGGACCTAGACTCGCG | 469/385 |
| DHN2 | VD2SF/VD2SR | CGATCTGATAGCAGCTCCAGC | CTCCTTTACCTCGCCTTCGTG | 472/288 |
| DHN3 | VD3SF/VD3SR | GCACCACCAGCATCATAGCAG | GGCACATTCAGTTGGAGTTGC | 433/288 |
| DHN4 | VD4SF/VD4SR | GAAGCGCGAGAGCAGTTCCA | AGGTGTGGTGGCCAGGTAGC | 401/275 |
| Actin | VActSF/VActSR | CTCTATATGCCAGTGGGCGTAC | CTGAGGAGCTGCTCTTTGCAG | 378/289 |

^*^ The first number indicates the length of pre-mRNA or unspliced mRNA in which the intron will not have been removed. The second number indicates the length of spliced, mature transcripts.
