# Supplementary material for: Identification of the dehydrin gene family from grapevine species and analysis of their responsiveness to various forms of abiotic and biotic stress
Source: BMC Plant Biol. 2012 Aug 10;12:140. doi: 10.1186/1471-2229-12-140 (PMC3460772; doi:10.1186/1471-2229-12-140)
Supplement: Additional file 6 — Sequence of primers used for real-time qRT-PCR in grapevine. [file 1471-2229-12-140-S6.doc]

**Additional file 6** Sequence of primers used for real-time qRT-PCR in grapevine.

| Gene | Primer pair | Forward primer sequence | Reverse primer sequence | Product (bp) |
| --- | --- | --- | --- | --- |
| DHN1 | VD1QF/VD1QR | CATGGGACGCAAGGACGAGC | GGACCTAGACTCGCGTTTCAG | 178 |
| DHN2 | VD2QF/VD2QR | AGAAACTGCCAGGACAGCACA | CTTCGGTCTTGGGGTGGTATC | 168 |
| DHN3 | VD3QF/VD3QR | GCACAAGGATGAATCAGTTCCAG | GCATGATCAGCCTCTTCACCT | 147 |
| DHN4 | VD4QF/VD4SR | GGGAAGCACAAGGAGGAGCA | AGGTGTGGTGGCCAGGTAGC | 172 |
| Actin | VActQF/VActQR | GTGCTGGATTCTGGTGATGGT | TCCCGTTCAGCAGTAGTGGTG | 167 |
